# Recent Progress in Photoelectrochemical Sensing of Pesticides in Food and Environmental Samples: Photoactive Materials and Signaling Mechanisms

**DOI:** 10.3390/molecules29030560

**Published:** 2024-01-23

**Authors:** Jie Song, Yuqi Chen, Ling Li, Mingqian Tan, Wentao Su

**Affiliations:** 1 State Key Laboratory of Marine Food Processing & Safety Control, Qingdao 266400, China; jiesongdpu@163.com; 2 State Key Laboratory of Marine Food Processing & Safety Control, National Engineering Research Center of Seafood, Academy of Food Interdisciplinary Science, School of Food Science and Technology, Dalian Polytechnic University, Qinggongyuan, Ganjingzi District, Dalian 116034, China; chenyuqi213@163.com (Y.C.); llingemail@163.com (L.L.); mqtan@dlpu.edu.cn (M.T.)

**Keywords:** photoelectrochemical sensing, pesticide residue, food and environmental safety, photoactive materials, signaling mechanisms

## Abstract

Pesticides have become an integral part of modern agricultural practices, but their widespread use poses a significant threat to human health. As such, there is a pressing need to develop effective methods for detecting pesticides in food and environmental samples. Traditional chromatography methods and common rapid detection methods cannot satisfy accuracy, portability, long storage time, and solution stability at the same time. In recent years, photoelectrochemical (PEC) sensing technology has gained attention as a promising approach for detecting various pesticides due to its salient advantages, including high sensitivity, low cost, simple operation, fast response, and easy miniaturization, thus becoming a competitive candidate for real-time and on-site monitoring of pesticide levels. This review provides an overview of the recent advancements in PEC methods for pesticide detection and their applications in ensuring food and environmental safety, with a focus on the categories of photoactive materials, from single semiconductor to semiconductor–semiconductor heterojunction, and signaling mechanisms of PEC sensing platforms, including oxidation of pesticides, steric hindrance, generation/decrease in sacrificial agents, and introduction/release of photoactive materials. Additionally, this review will offer insights into future prospects and confrontations, thereby contributing novel perspectives to this evolving domain.

## 1. Introduction

Pesticides are a general term for chemicals for preventing, eliminating, and controlling diseases caused by pests, insects, and weeds [1]. The application of pesticides significantly contributes to the advancement of agricultural production to meet the world’s increasing population [2]. Based on their effects, pesticides include several types, like herbicides, insecticides, fungicides, and rodenticides [3]. According to their chemical structures, pesticides can be classified into carbamate, neonicotinoid, organophosphate, organochlorine, and pyrethroid [4]. Most of the pesticides are hazardous, and their excessive or improper use may cause pesticide residue in crops, the atmosphere, soil, and water resources, posing a severe threat to the environmental system. Moreover, pesticide residue can enter the human body through the food chain to cause chronic or acute poisoning [5,6,7,8,9], manifesting as an increased risk of cancer, debilitating illnesses, and severe or even fatal symptoms [10,11,12,13,14]. In recent years, pesticide residues have aroused global concern as a significant issue in environmental and food safety [15]. Many countries have established policies in terms of their national conditions to stipulate the maximum residue limits of pesticides in agricultural products and acceptable daily intake from food [16,17]. Therefore, effective detection of pesticides in food and environmental samples is a crucial step in implementing these policies and ensuring the health of human beings [18,19].

To date, a variety of methods have been developed for pesticide detection. Chromatography methods, including gas chromatography (GC) [20], high-performance liquid chromatography (HPLC) [21], and chromatography-mass spectrometry (LC-MS, HPLC-MS), exhibit high accuracy and sensitivity and are regarded as the gold standard detection approach to the analysis of pesticide residue levels [22,23]. However, the demand for sophisticated and expensive instruments, laborious sample pretreatment, and professional operators limits their on-site application [24]. Common rapid detection methods such as capillary electrophoresis [25], fluorescence [26], enzyme-linked immunosorbent assay [27], and surface-enhanced Raman scattering [28] have satisfactory sensitivity and selectivity but still suffer from short storage times and solution instability [19,29]. As a consequence, there still need to be simple, low-cost, highly accurate, and sensitive methods for the sake of real-time and on-site monitoring of pesticide levels [30,31].

Photoelectrochemical (PEC) sensing is a newly emerged technique developed on the basis of electrochemical sensing via a systematic integration of light excitation and electrochemical analysis [32,33,34,35]. By using electrochemical instruments to output signals, PEC sensing technology inherits the salient features of low cost, simple operation, fast response, capability of real-time monitoring, and easy miniaturization of instruments from the traditional electrochemical detection method [36,37,38]. Moreover, due to the complete separation of light excitation and signal output, the PEC sensing method has much lower background signals than those of the conventional electrochemical method [39,40,41]. Also, the strong redox ability of photogenerated charges reduces the dependence on the applied potential, thus endowing PEC sensing superior performance compared to electrochemical analysis [42]. Owing to these advantages, PEC sensing has been widely used in many fields such as disease diagnosis [43,44,45], health monitoring [46,47,48], and medical science [49,50]. Meanwhile, it meets the demand for rapid, convenient, sensitive, and accurate detection of pesticide residues in food and environmental samples [41,51,52].

**Figure 1:**
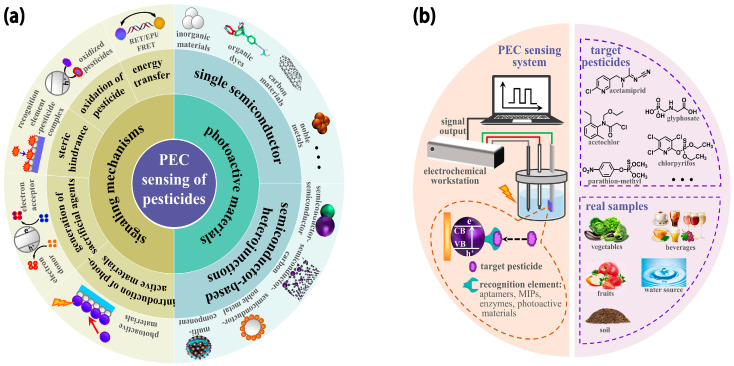
Schematic illustration of the building blocks of PEC sensing of pesticides: (**a**) photoactive materials and signaling mechanisms; (**b**) PEC sensing systems, targets, and real samples.

Currently, several review reports have been published focusing on the advancement of PEC sensing technologies and their applications to multifarious target analytes [34,35,36,37,41]. Nevertheless, an integral overview of PEC sensors concentrated on pesticide analysis for food and environmental safety is rare. We have witnessed inspiring progress in the PEC sensing of pesticides with high accuracy and sensitivity based on various efficient photoactive materials and flexible recognition mechanisms in recent years. Thus, this work presents a systematic and comprehensive review of the achievements in PEC sensing of pesticides. As displayed in Figure 1a, we intend to provide the readers with a clear view of the PEC sensors for pesticide detection, ranging from photoactive materials to signaling mechanisms. At the same time, the challenges and prospects in this area are also put forward.

## 2. Principles of the PEC Sensing System

A PEC sensing system converts light energy to electrical signals (Figure 1b), and its detection principle can be generally described as follows: Under illumination with higher energy than its band gap, the photoactive material on the photoelectrode absorbs photons and produces photogenerated electron-hole (e^−^-h^+^) pairs. For n-type semiconductors, electrons in the conduction band (CB) migrate to the photoelectrode, while holes in the valence band (VB) are neutralized by an electron donor in the electrolyte, generating an anode photocurrent. For p-type semiconductors, electrons donated from the photoelectrode neutralize the holes in the VB, while CB electrons transfer to the electron acceptor in the electrolyte, producing a cathode photocurrent. When recognition elements in the PEC sensing system interact with the target analyte, the PEC response of the photoactive material changes and exhibits a certain correlation with the concentration of the analyte [53,54,55,56]. There are two types of recognition elements for PEC sensing of pesticides: the first type includes aptamers, enzymes, and molecular imprinted polymers (MIPs), which are fixed on the photoelectrode surface of the photoactive materials via chemisorption, noncovalent/covalent binding, and affinity binding; the other type is the photoactive materials mobilized on the photoelectrode surface, which can directly react with pesticide to form a complex or cause a redox reaction.

## 3. Photoactive Materials

Photoactive material selection and material properties are key elements that impact the performance of the PEC sensor [57,58]. Almost all the PEC sensing platforms for different analyte detections (e.g., biomolecule monitoring, antibiotic detection, pesticide sensing) follow the same principle: photoactive material with high e^−^-h^+^ separation efficiency displays excellent PEC conversion ability, which will contribute to the high sensitivity of the PEC sensor [59,60]. Up to now, various semiconductors with photoactivity have been developed and employed in the construction of PEC sensing systems. This section will provide an overview of the categories of photoactive materials, including single semiconductors and semiconductor-based heterojunctions, along with an introduction to their PEC properties, which is a precondition for developing effective strategies for the detection of pesticides.

### 3.1. Single Semiconductor

Semiconductor element-based photoactive materials have been widely investigated as PEC transducers. Some of them have wide band gaps and are ultraviolet (UV) sensitive, such as TiO_2_, ZnO, ZnS, BiOCl, etc. [61,62,63,64]. Due to the damaging effect on organic targets and biomolecules caused by high-energy UV illumination, some semiconductors with narrow band gaps and visible-responsive properties are being developed for PEC sensing, including CdX (X = S, Se, Te), PbS, Bi_2_S_3_, BiVO_4_, etc. [65,66,67,68,69]. Noble metals such as gold and silver with various nanostructures are also regarded as photoactive materials owing to their discrete band gaps and size-dependent PEC properties [70,71]. Inspired by organic dye-sensitized solar cells (DSSCs), some organic dyes with rational-designed structures, for example, porphyrin [72], triphenylamine [73], and ruthenium bipyridyl derivatives [74], have been employed as both photoactive materials and recognition elements in PEC sensors. Recently, two-dimensional layered materials including graphene, graphitic phase carbon nitride (g-C_3_N_4_), MXenes, metal-organic frameworks (MOFs), and covalent organic frameworks (COFs) have become research hotspots for designing PEC sensors due to their large specific surface areas, excellent charge mobility, and outstanding stability [48,75,76,77,78,79,80]. 

**Figure 2:**
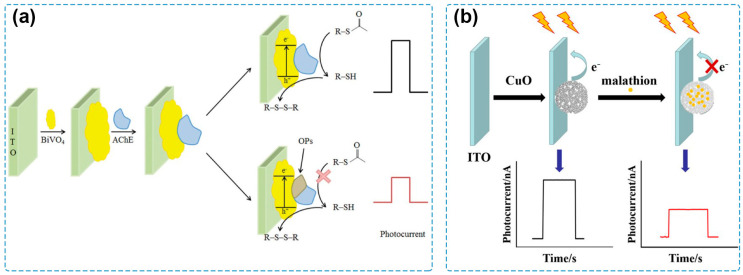
(**a**) Schematic of a PEC sensor for chlorpyrifos detection by using an ITO/BiVO_4_ photoelectrode [87]. Copyright 2021 Taylor & Francis. (**b**) Schematic of a PEC sensor for malathion detection based on Cu-BTC MOF-derived CuO material [88]. Copyright 2019 Springer Nature.

In the early stages of the development of PEC sensing, classical single-component semiconductors were used as photoactive materials for the tentative exploration of PEC sensing technology. For example, a PEC sensing system for detecting acetylcholinesterase (AChE) activity and its inhibitor by employing CdS quantum dots (QDs) to construct the photoelectrode is one of the pioneering works in the history of PEC sensing [81]. Afterwards, PEC sensors based on CdSe QDs, CdTe QDs, ruthenium bipyridyl derivatives, and porphyrin derivatives were used for the detection of several kinds of target analytes, including small biomolecules, cells, and heavy metal ions [66,82,83,84]. With the progress in material preparation and photoelectrode modification, other semiconductors were synthesized and applied for PEC sensing of pesticides. A PEC sensor based on polythiophene derivative film (PS_2_TTz) via a one-step electropolymerization process was constructed by Xu et al. [85], where PS_2_TTz serves both as a photoactive material and a recognition element for chlorpyrifos, displaying a detection range from 1 to 218.92 μg/L and a limit of detection (LOD) of 0.36 μg/L. Crossed BiOI nanoflake arrays were immobilized on an indium tin oxide (ITO) glass substrate via successive ionic layer adsorption and reaction approaches as a photocathode to detect methyl parathion, a model organophosphate pesticide [86]. By coupling with AChE as the recognition element, the fabricated PEC system showed an LOD of 0.04 ng/mL and fine applicability for the detection of spiked food samples, including garlic, apple, and cabbage. Miao and coworkers investigated the effect of treatment temperature on the photocurrent response of BiVO_4_ and reported a sensitive PEC biosensor for pesticide detection by using the ITO/BiVO_4_ photoelectrode [87] (Figure 2a). Based on the best PEC performance obtained after the treatment at 300 °C, the PEC sensor could detect chlorpyrifos with an LOD of 0.25 pM and showed satisfactory recovery values for the analysis of spiked real-life samples, including grape, strawberry, spinach, and water samples. By calcination of Cu-benzene-1,3,5-tricarboxylic acid (Cu-BTC) MOF at 300 °C, a hierarchical CuO material was obtained and modified on an ITO electrode [88] (Figure 2b), possessing a high PEC conversion efficiency under visible-light irradiation. Based on the formation of the CuO–malathion complex, the as-prepared PEC sensor had an LOD of 8.6 × 10^−11^ M and a successful application for the analysis of spiked Chinese cabbage samples.

### 3.2. Semiconductor-Based Heterojunctions

Despite some single-component photoactive materials that have been reported, the drawbacks of limited light-absorption ability, easy recombination of photogenerated e^−^-h^+^ pairs, or serious photo-corrosion have impeded their PEC conversion efficiency and confined their practical application to a great extent [35,38,41]. To improve the PEC properties of the photoactive materials and to enhance the performance of the PEC sensors, heterojunctions are favorable for the construction of PEC sensing systems.

#### 3.2.1. Semiconductor–Semiconductor Heterojunction

**Figure 3:**
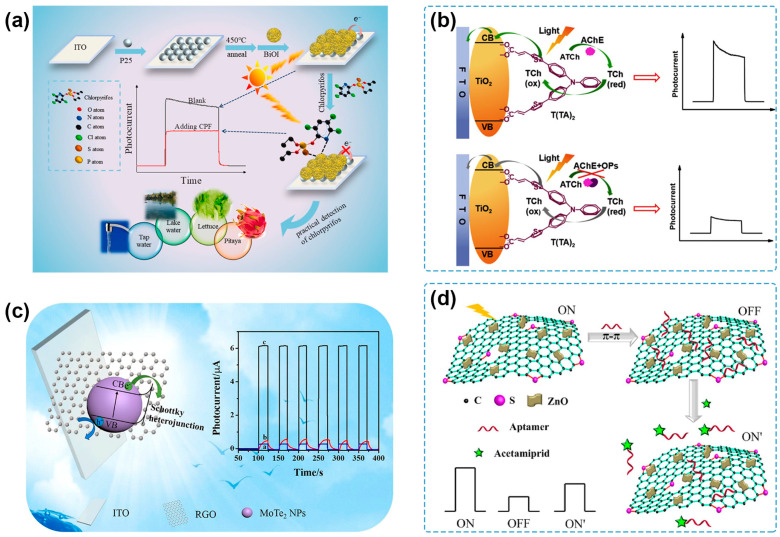
(**a**) Schematic illustration of the PEC sensing platform for the detection of chlorpyrifos based on BiOI/AR-TiO_2_ heterojunction [93]. Copyright 2023 Elsevier. (**b**) Schematic illustration of PEC analysis of parathion based on T(TA)_2_-TiO_2_ modified photoelectrode [98]. Copyright 2018 Elsevier. (**c**) PEC performance enhancement mechanism diagram and photocurrent of RGO-curve a, MoTe_2_ NPs-curve b, and MoTe_2_ NPs/RGO-curve c based on MoTe_2_ NPs/RGO heterostructure [99]. Copyright 2021, Elsevier. (**d**) Schematic representation for PEC acetamiprid aptasensor based on t-SG/ZnO heterojunction [100]. Copyright 2017 Elsevier.

It has been considered an effective approach to constructing heterostructures based on two semiconductors with different band gaps to acquire photoelectrodes with the desired PEC properties [29]. The most typical heterojunction used in PEC sensing is composed of two kinds of inorganic semiconductors with suitable energy levels for e^−^-h^+^ pair separation. TiO_2_ is one of the classical photoactive materials used in PEC sensing due to its good biocompatibility and photochemical stability [89]. However, its wide band gap results in limited excitation only under UV light, which is known for harming biological substances and having a low proportion in natural light [90,91]. Zhou and coworkers synthesized a nanocomposite of g-C_3_N_4_ and TiO_2_ nanotube arrays (TNA/g-C_3_N_4_) by in situ generating g-C_3_N_4_ on the surface of TNA [92]. Due to the relatively narrow band gap of g-C_3_N_4_ and the suitable energy level matching between TNA and g-C_3_N_4_, TNA/g-C_3_N_4_ showed a photocurrent response in the visible-light region. By utilizing TNA/g-C_3_N_4_ as a photoelectrode, a PEC sensor for acetamiprid detection was fabricated with the combination of λ exonuclease-assisted recycling amplification and DNAzyme-catalyzed precipitation, showing a low LOD of 0.025 pM and application for the analysis of spiked tomato, cucumber, and bitter gourd samples. Lyu and coworkers developed an n–n heterojunction by immobilizing BiOI on the surface of TiO_2_, which had a heterophase junction of anatase/rutile (AR-TiO_2_) [93] (Figure 3a). The different functions of AR-TiO_2_ built an internal electric field to promote carrier transfer across the heterophase, and the three-dimensional BiOI had a loose porous structure consisting of abundant small and thin nanosheets, which contributed to enhanced light absorption and separation of photogenerated e^−^-h^+^ pairs. The PEC sensor showed good performance toward detecting chlorpyrifos with an LOD of 0.24 pg/mL and a potential application value for detecting spiked lettuce, pitaya, and water samples. Bi_2_S_3_@g-C_3_N_4_, a p–n heterojunction photoactive composite, was prepared by Lei et al. to promote charge transfer and suppress e^−^-h^+^ pair recombination [94]. The obtained Bi_2_S_3_@g-C_3_N_4_/ITO photoelectrode was used to detect chlorpyrifos based on the affinity between chlorpyrifos and the Bi (III) surface, presenting a linear range from 50 ng/mL to 0.1 mg/mL and an LOD of 0.03 ng/mL. Furthermore, the novel PEC sensing platform could support fast and effective monitoring of spiked green vegetable samples.

Organic molecule-based photovoltaic materials have attracted much attention for their high visible-light absorption, precise molecular structures, and controllable assembling process. Therefore, the organic–inorganic heterojunction can lay a solid foundation for PEC sensing and pesticide detection. Poly(3-hexylthiophene) (P3HT) is one of the excellent photoelectric materials with high charge carrier mobility and strong absorption in the visible region [95]. Hu’s group used P3HT as a sensitizer of TiO_2_ to prepare P3HT/TiO_2_ nanocomposites for the PEC detection of chlorpyrifos [96]. Later, they reported a TiO_2_-P3HT-ionic liquid (IL) nanocomposite film by blending TiO_2_ nanoparticles (NPs) and P3HT at room temperature IL [97]. The improved ionic conductivity resulted from IL was beneficial for promoted charge transfer and higher PEC efficiency, and the proposed PEC sensor could detect acetochlor with an LOD of 0.2 nM. The results of spiked water samples were in acceptable agreement with those of the GC-MS method. Song and coworkers synthesized a di-branched di-anchoring dye, T(TA)_2_, to sensitize TiO_2_ NPs for the highly sensitive PEC sensing of organophosphate pesticides [98] (Figure 3b). The unique D-(π-A)_2_ structure endows the T(TA)_2-_TiO_2_ nanocomposites with higher PEC conversion efficiency and superior photo-stability in aqueous solutions, even better than two kinds of organic dye-sensitized TiO_2_ nanocomposites mostly used in DSSCs or PEC sensing fields. The PEC sensor showed a wide linear range of 2 × 10^−12^–4 × 10^−6^ g/mL, a low LOD of 5.6 × 10^−13^ g/mL, and acceptable accuracy for the detection of spiked apple juice samples. 

#### 3.2.2. Semiconductor–Carbon Heterojunction

Carbon-based materials have diverse electronic structures of C elements (sp, sp^2^, and sp^3^ hybridization) and various nanostructures, including nanosheets (NSs), nanofibers (NFs), and nanotubes (NTs) [101,102,103]. Due to their inherent merits, such as good chemical stability, fast electron mobility, excellent biocompatibility, and large specific surface area, carbon materials have been extensively employed for coupling with semiconductors to obtain heterojunctions for developing PEC sensors. They are usually considered ideal electron transfer media to accelerate the transfer of photogenerated electrons and promote the separation of e^−^-h^+^ pairs [38,41]. Additionally, a large surface area can also provide abundant active sites to absorb electroactive species and biomolecules via covalent bonding, π–π packing, and hydrogen bonding [104]. Therefore, semiconductor–carbon heterojunction usually contributes to the better PEC properties of photoactive materials and endows PEC sensors with superior performance. 

Graphene (GR) and reduced graphene oxide (RGO) are the most common carbon materials used for constructing semiconductor–carbon heterojunctions in PEC sensing of pesticides. BiPO_4_-functionalized RGO nanocomposites (BiPO_4_-RGO) were synthesized and showed enhanced PEC response compared with the pure BiPO_4_ NPs. Because the photogenerated electrons can be stored by RGO NSs, the accelerated electron transfer reduced the recombination rate of the photogenerated e^−^-h^+^ pairs [105]. The as-developed PEC sensor for chlorpyrifos detection exhibited a broad linear range from 0.05 to 80 ng/mL, a low LOD of 0.02 ng/mL, and satisfactory results according to the determination of spiked wastewater samples. Ding and coworkers fabricated a MoTe_2_ NPs/RGO heterostructure via a one-step hydrothermal synthesis process [99] (Figure 3c). Due to the suitable Schottky barrier, the obtained heterostructure exhibited a photocurrent intensity of 21.8 times that of RGO and 10.5 times that of MoTe_2_ NPs, respectively. Then, a label-free PEC aptasensor was successfully constructed to determine profenofos in concentrations ranging from 10^−9^ to 10^−2^ g/L with an LOD of 3.3 × 10^−10^ g/L and was successfully conducted in spiked vegetable samples. p-type BiOBr nanoplates decorated with n-type N-doped GR (BiOBr-NGR) composites were prepared to construct a p–n heterojunction for the separation of photogenerated e^−^-h^+^ pairs and enhanced PEC response [106]. Based on the improved photocurrent signal, a PEC sensor was developed for the detection of chlorpyrifos, presenting a low LOD of 1.67 pg/mL and offering a general approach to chlorpyrifos detection in food and environmental samples. Yan and coworkers fabricated a thiophene-sulfur-doped GR/ZnO nanocomposite (t-SG/ZnO) [100] (Figure 3d), where the t-SG/ZnO had a narrower band gap, stronger visible-light absorption, and better photo-stability than that of the pure ZnO nanoplates because of the facilitated interfacial charge transfer. Meanwhile, t-SG/ZnO also exhibited a PEC response of about 1.5 times and 2.6 times that of N-doped GR/ZnO and GR/ZnO, respectively. On this basis, an “on-off-on” PEC aptasensor was constructed for the detection of acetamiprid, showing an LOD of 0.33 ng/mL and analytical applicability for the detection of spiked cucumber samples. 

#### 3.2.3. Semiconductor–Noble Metal Heterojunction

Noble metals, including gold, silver, and platinum, have excellent conductivity and strong electron trapping abilities, so they can serve as electron traps to accelerate the transportation of charge carriers and suppress the recombination of the photogenerated e^−^-h^+^ pairs [107,108,109]. Generally, there are two possible paths for the electron transfer in a semiconductor–noble metal heterojunction [110]: in one situation, the photoexcited electrons from the semiconductor will transfer to the noble metals, forming an energy transfer process; in the other situation, when the heterojunction is composed of nanostructured noble metal and wide band gap semiconductor, hot electrons induced by the surface plasmon resonance (SPR) effect will transfer from the noble metal to the semiconductor, along with enhanced visible-light absorption at the same time. 

**Figure 4:**
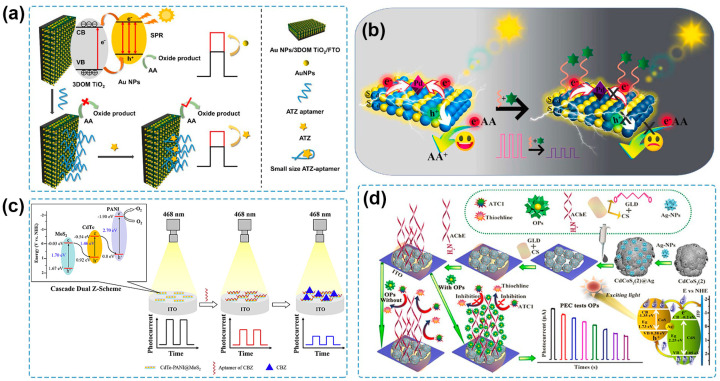
(**a**) Schematic illustration of an atrazine PEC sensor based on an Au NPs/3DOM TiO_2_ photoanode [112]. Copyright 2023 Elsevier. (**b**) Schematic illustration of the carbendazim PEC aptasensor based on Pd NPs/CdS/ITO [113]. Copyright 2022, Elsevier. (**c**) Schematic illustration of PEC aptasensor for the detection of carbendazim based on cascade dual Z-scheme CdTe-PANI@MoS_2_ [114]. Copyright 2023 Elsevier. (**d**) Schematic illustration of a PEC biosensor for chlorpyrifos detection based on a CdCoS_2_(2)@Ag photoelectrode [115]. Copyright 2022 Elsevier.

Recently, Au NPs have been frequently coupled with nanostructured TiO_2_ to establish PEC sensing systems for pesticide detection. A PEC aptasensor was fabricated for the detection of diazinon by using Au NPs/TiO_2_ nanocomposite as the photoactive material [111]. The use of Au NPs not only improved the PEC properties of the Au NPs/TiO_2_ nanocomposite with a higher photocurrent response compared to the pure TiO_2_, but also provided active sites for the aptamer conjugating to the Au NPs/TiO_2_ through S-Au bonds. The as-fabricated PEC sensor showed high sensitivity, selectivity, reproducibility, and stability for the quantitative determination of diazinon and obtained satisfactory results for the detection of diazinon in water and biological samples. Zhang and coworkers developed a PEC sensing platform based on Au NPs modified with a three-dimensionally ordered macroporous (3DOM) TiO_2_ nanostructure frame for trace detection of atrazine [112] (Figure 4a). Owing to multi-signal amplification of the 3DOM structure of TiO_2_ and the SPR effect of Au NPs, the Au NPs/3DOM TiO_2_ photoanode showed improved PEC performance under visible light. Meanwhile, its orderly nanostructure contributed to a large packing density of aptamers by Au-S bond with dominant spatial orientation for the specific recognition of atrazine, thus providing the PEC aptasensor with a low LOD of 0.167 ng/L and a great application prospect based on analysis of real water samples. Besides Au NPs, Pd NPs are another plasmonic nanostructured noble metal used to construct semiconductor–noble metal heterojunctions for pesticide determination. Wen and coworkers prepared Pd NPs decorated CdS microsphere (Pd NPs/CdS) for the PEC aptasensing of carbendazim [113] (Figure 4b). Based on the SPR effect of Pd NPs and the Schottky junction between Pd NPs and the CdS microsphere, the photocurrent obtained from Pd NPs/CdS was 7.7 times higher than that of the bare CdS microsphere. The as-fabricated PEC aptasensor possessed excellent performance with a wide linear range of 1.0 × 10^−12^–1.0 × 10^−6^ M and a low LOD of 3.3 × 10^−13^ M. Furthermore, the PEC aptasensor was employed for the analysis of spiked lettuce samples with satisfactory results.

#### 3.2.4. Multicomponent Heterojunction

A multicomponent heterojunction is composed of semiconductors, carbon materials, or noble metals with more than two components. It involves interactions between several materials and usually consists of more than one heterojunction, thus combining the advantages of different types of heterojunctions and providing the opportunity to elaborately match the band levels of each semiconductor for an optimized electron transfer path [36]. For example, a TiO_2_/S-BiVO_4_@Ag_2_S heterojunction with cascade band-edge levels can accelerate charge transfer and promote separation of the charge carriers effectively, presenting a photocurrent intensity about 2.1 times higher than that obtained from the TiO_2_/S-BiVO_4_ electrode [116]. A CuO/Pd NPs nanocomposite coupled with CdS QDs can exhibit a synergistic effect through Schottky junction and sensitization, therefore suppressing efficient charge recombination and light absorption to a great extent [117].

Recently, considerable efforts have been devoted to designing and constructing multicomponent heterojunctions for PEC sensing of pesticides. Zhou and coworkers prepared a BiOI-Bi/short carbon nanotubes (BiOI-Bi/s-CNTs) composite that contained a semiconductor–metal heterojunction and a semiconductor–carbon heterojunction [118]. An intensive photocurrent response was achieved due to the active SPR effect of Bi NPs and the high charge mobility of s-CNTs, and the PEC sensor was established for chlorpyrifos detection, showing an LOD of 1.89 pg/mL and satisfactory recoveries of chlorpyrifos in spiked cabbage and Chinese cabbage samples. A PEC aptasensor for carbendazim detection was constructed based on the CdTe-polyaniline@MoS_2_ heterostructure [114] (Figure 4c). The cascade Z-scheme electron transfer resulted in a significantly increased photocurrent, which was 2.7-fold higher than that at CdTe/MoS_2_ for the facilitated separation of photogenerated e^−^-h^+^ pairs. The fabricated PEC aptasensor possessed a linear range of 0.1–100 ng/mL with an LOD of 0.033 ng/mL, and it was successfully applied for spiked tomato sample analysis. Zheng and coworkers synthesized a porous hollow CdCoS_2_(2) microsphere consisting of a CdS-CoS heterojunction with well-matched energy bands and outstanding PEC properties, which was further improved by compositing CdCoS_2_(2) with the conductive Ag NPs [115] (Figure 4d). The PEC sensor was used to monitor chlorpyrifos based on its inhibition effect on AChE and achieved a wide linear response range (0.001 to 270 μg/mL), a low LOD (0.57 ng/mL), and successful application for the chlorpyrifos determination in spiked river water samples.

## 4. Signaling Mechanisms

Almost all PEC sensors achieve selective detection of the target based on specific recognition between the target and the recognition element. As an important application area of PEC sensing, PEC sensors for pesticide detection share common recognition element categories and signaling transduction strategies with PEC sensing platforms for the detection of other small molecules. For pesticide detection, recognition elements include aptamers [92], enzymes [98,115], MIPs [119], and photoactive materials [88,93], and their chemical compositions and molecular structures are usually selected or designed according to the specific target pesticides. The reactions between the recognition element and the target pesticide will subsequently trigger physical or chemical reactions and cause the photocurrent change of the PEC sensor via a series of signaling mechanisms. This section will provide an overview of the signaling mechanisms for PEC sensing of pesticides, which are important for the design of recognition strategies for the PEC sensors. 

### 4.1. Oxidation of Pesticide

During the PEC sensing process, photoactive materials are excited under illumination to generate e^−^-h^+^ pairs, and the holes with strong oxidative power can directly oxidize some target pesticides or oxidize some hole mediators to form free radical moieties, which further induce the oxidation of the target pesticides. Such interaction between the photogenerated holes and the pesticides is beneficial for the neutralization of the holes and the separation of the photogenerated e^−^-h^+^ pairs, thus improving the PEC conversion efficiency and enhancing the photocurrent response [120].

Wang and coworkers fabricated a PEC sensor based on Au NPs and poly(*o*-phenylenediamine) (PoPD)-modified TiO_2_ NTs for the detection of lindane [119]. PoPD not only served as an MIP to specifically capture lindane molecules but also acted as a photoactive material to generate charge carriers under visible-light excitation. The electrons transferred from the lowest unoccupied molecular orbital of PoPD to the CB of the TiO_2_ NTs, while the holes of PoPD were consumed by taking part in the oxidation of the lindane, promoting the separation of the photogenerated e^−^-h^+^ pairs and the amplification of the photocurrent response. A similar work was proposed by the same research group for the PEC sensing of chlorpyrifos, in which a PoPD-AuNPs/TiO_2_ NTs composite was proposed by using Au NPs to improve the interfacial charge transfer efficiency [121]. Based on the oxidation of the chlorpyrifos by photogenerated holes, the photocurrent increased proportionally to chlorpyrifos levels ranging from 0.05 to 10 μM, with an LOD of 0.96 nM. Furthermore, the PEC sensor was successfully applied to the determination of spiked green vegetables. 

**Figure 5:**
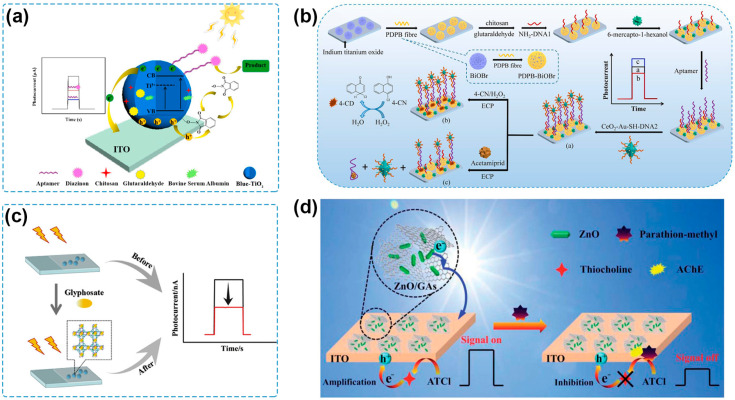
(**a**) Schematic illustration of a PEC aptasensor based on /B-TiO_2_/ITO with NHPI as the hole mediator [123]. Copyright 2022, Elsevier. (**b**) Schematic illustration of the PEC aptasensor based on poly (diphenylbutadiene)-BiOBr heterojunction and Au-modified CeO_2_ octahedrons [127]. Copyright 2022, Elsevier. (**c**) Schematic illustration of a Cu-BTC/CN-NS-based PEC sensor for glyphosate detection [135]. Copyright 2019 Elsevier. (**d**) Schematic illustration of the PEC biosensor with ZnO/GAs nanocomposites based on the inhibition effect of AChE [146]. Copyright 2021, Royal Society of Chemistry.

When a hole mediator exists in the PEC sensing system, it can be oxidized by the photogenerated holes to form free radicals with superior oxidative abilities, which can subsequently oxidize the pesticides. H_2_O is the most commonly used hole mediator, as PEC sensing measurements are often carried out in aqueous solutions. For example, a PEC sensor for chlorpyrifos determination was fabricated based on a 1D TiO_2−x_/3D nitrogen-doped graphene hydrogel (NGH) heterostructure [122]. Under light excitation, the photogenerated holes were clustered on the VB of TiO_2_ and could oxidize H_2_O to produce hydroxyl radicals (•OH). Then, •OH and holes induced the oxidation of chlorpyrifos, leading to an amplified photocurrent response. The constructed PEC sensor possessed high reliability and accuracy for chlorpyrifos detection and exhibited good performance for practical determination in wastewater samples. Wen and coworkers developed a PEC sensing platform by employing Blue-TiO_2_ (B-TiO_2_) and *N*-hydroxyphthalimide (NHPI) as the photoactive material and the hole mediator, respectively [123] (Figure 5a). Photogenerated holes transferred from the VB of B-TiO_2_ to NHPI and oxidized NHPI to phthalimide-*N*-oxyl radical, which could oxidize the diazinon captured by the aptamer immobilized on the photoelectrode surface. The as-fabricated PEC aptasensor exhibited a linear range for diazinon detection from 0.1 to 1000 nM with an LOD of 0.03 nM and showed food recovery results for spiked apple samples.

### 4.2. Steric Hindrance

Steric hindrance resulting from molecular recognition reactions is another signaling strategy in the construction of PEC sensing platforms. For pesticide detection, the common recognition reactions consist of biocatalytic precipitation and binding interactions between the pesticide and the recognition elements. The introduction of steric hindrance may lead to diffusion suppression of electron donor/acceptor to photogenerated carriers and transfer inhibition of the photogenerated carriers to the photoactive materials, thereby resulting in a significant decrease in photocurrent response [55].

Biocatalytic precipitation (BCP) is an effective approach to inducing steric hindrance to the PEC sensing systems. Horseradish peroxidase (HRP) can facilitate the oxidation of 4-chloro-1-naphthol (4-CN) by H_2_O_2_ to generate benzo-4-chlorohexadienone (4-CD), an insoluble and insulating precipitate, on photoelectrode surfaces [124]. Based on this principle, some DNAzymes or nanozymes have been designed to simulate the activity of HRP for a BCP reaction [92,125,126,127]. Zhou and coworkers developed a PEC aptasensor for acetamiprid detection. Combining aptasensing and λ exonuclease-assisted recycling amplification, an HRP-mimicking hemin/G-quadruplex DNAzyme formed and biocatalytically oxidized 4-CN to generate 4-CD, inhibiting electron donor diffusion to the surface of the photoanode and reducing the photocurrent response [92]. A PEC aptasensor was constructed by using CeO_2_-Au nanozyme as a signal amplification platform to conduct a BCP reaction and quench the photocurrent signals [127] (Figure 5b). The recognition of target acetamiprid caused the release of CeO_2_-Au from the photoelectrode, thus weakening the quenching effect and recovering the photocurrent signal. The obtained PEC aptasensor exhibited an LOD of 0.05 pM over a linear range of 0.1 pM–10 μM acetamiprid and a high acetamiprid detection accuracy in spiked cucumber samples.

The specific binding of the recognition element to the pesticide is another strategy to form steric hindrance on the photoelectrode surface. For example, when a target pesticide is captured by its aptamer, or MIP, the formation of an aptamer–pesticide complex or MIP–pesticide complex will increase the steric hindrance and decrease the conductivity for electron transfer [128,129,130,131,132,133,134]. A PEC sensor was constructed by Cao and coworkers based on a hierarchically porous Cu-BTC/g-C_3_N_4_ nanosheet (Cu-BTC/CN-NS) as both a photoactive material and the recognition element for glyphosate [135] (Figure 5c). During the PEC sensing process, the Cu metal center coordinated with glyphosate to form Cu–glyphosate complexes, resulting in an increased steric hindrance to block electron transfer and an obvious decrease in photocurrent. The constructed PEC sensor can achieve the detection of glyphosate with a low LOD (1.3 × 10^−13^ M) and a wide detection range (1.0 × 10^−12^–1.0 × 10^−8^ M and 1.0 × 10^−8^–1.0 × 10^−3^ M), and it also showed great analytical performance in PEC sensing of glyphosate in spiked soybean samples. A similar strategy was utilized for the PEC sensing of malathion, exhibiting high sensitivity and potential application in real-life sample analysis [88]. Besides Cu-containing photoactive materials, some photoactive materials, including Bi and Co-based semiconductors, can also form similar photoactive material–pesticide complexes, which contribute to increased steric hindrance, suppressed charge transfer, and reduced photocurrent response [93,105,106,135,136,137,138,139,140,141].

For PEC sensing platforms based on the steric hindrance strategy, the conductivity of the photoelectrode interface decreases as the pesticide concentration increases. Meanwhile, long operation times might promote the formation of recognition element–target pesticide complexes and contribute to the steric hindrance. To achieve high sensitivity, some researchers optimized the incubation or adsorption time until the binding reactions reached saturation [128,131]. As the steric hindrance is caused by the formation of recognition element–target pesticide complexes, most of the sensing photoelectrodes are not reusable due to the irreversible binding reactions between the recognition elements and the target pesticides. Only some PEC sensors using MIPs as recognition elements mentioned that the molecularly imprinted electrode could be reused several times by eluting the templates to regenerate the MIPs [132,142,143].

### 4.3. Generation/Decrease in Sacrificial Agents

Regulating the generation of the sacrificial agents is a straightforward approach to designing the signaling strategy of a PEC sensor. On one hand, the in situ generated electron donor/acceptor can react with excited holes/electrons, thus suppressing the recombination of the photogenerated e^−^-h^+^ pairs to obtain increased and stable photocurrent signals [37]. A simple example is the hydrolyzation of parathion-methyl in alkaline conditions, and the hydrolysate, p-nitrophenol, which served as the electron donor, is oxidized by the holes to amplify the photocurrent response [144,145]. On the other hand, the inhibition of the generation of sacrificial agents will in turn decline the PEC signal. 

Enzymatic reactions are commonly utilized to establish the signaling mechanism for the PEC sensing of organophosphate pesticides. AChE is a typical enzyme used in a PEC sensor for pesticide detection due to the catalytical hydrolysis of acetylthiocholine (ATCh) to generate thiocholine (TCh) as electron donors, resulting in an enhanced photocurrent response [98]. Organophosphate pesticides can inhibit the bioactivity of AChE, and the photocurrent signal will decrease due to the reduced production of TCh [115]. Upon this method, some PEC sensing platforms for organophosphate pesticides have been developed by collaborating photoactive materials with high PEC conversion efficiency [98,115,146,147]. For example, a self-powered PEC sensor based on ZnO nanorod/3D graphene aerogel-sensitized structure and inhibition effects of parathion-methyl on AChE was fabricated (Figure 5d), showing the detection of parathion-methyl with a linear range of 0.1 ng/mL to 0.1 μg/mL and a LOD of 0.03 ng/mL. Cheng and coworkers proposed a visible-light-driven and self-powered PEC sensor based on AChE-immobilized N-doped carbon QDs/TiO_2_/ITO photoelectrode [147], which can achieve the detection of chlorpyrifos with high sensitivity (a LOD of 0.07 ng/mL), board detection range (0.001–1.5 μg/mL), and accurate determination of spiked lake water and Chinese cabbage samples. It should be noticed that once removed from the pesticide solution, the AChE-modified photoelectrodes cannot regain their enzymatic activity due to the irreversible inhibition of pesticides, and thus the photoelectrodes are single-use items in theory. The inhibited AChE can be reactivated when using nucleophilic compounds such as pralidoxime iodide. For example, Du and coworkers have observed that an AChE-modified electrode inhibited by malathion could recover more than 90% of its original activity after immersing in a pralidoxime iodide solution for several minutes [148], which might be helpful to obtain reusable photoelectrodes. Apart from AChE, other enzymes, including alkaline phosphatase and glucose oxidase, have been employed on account of the catalytical hydrolysis of their substrates to in situ generate ascorbic acid and H_2_O_2_, serving as electron donors to enhance the PEC conversion efficiency and amplify the photocurrent response [149,150].

### 4.4. Introduction/Release of Photoactive Materials

The introduction/release of photoactive species is an effective method for PEC signaling. By using the recognition event to introduce/release the photoactive materials on/from the photoelectrode, the PEC properties, including light absorption properties, e^−^-h^+^ generation, and charge transfer, can be regulated, thereby leading to the change of PEC signals [37,38]. 

**Figure 6:**
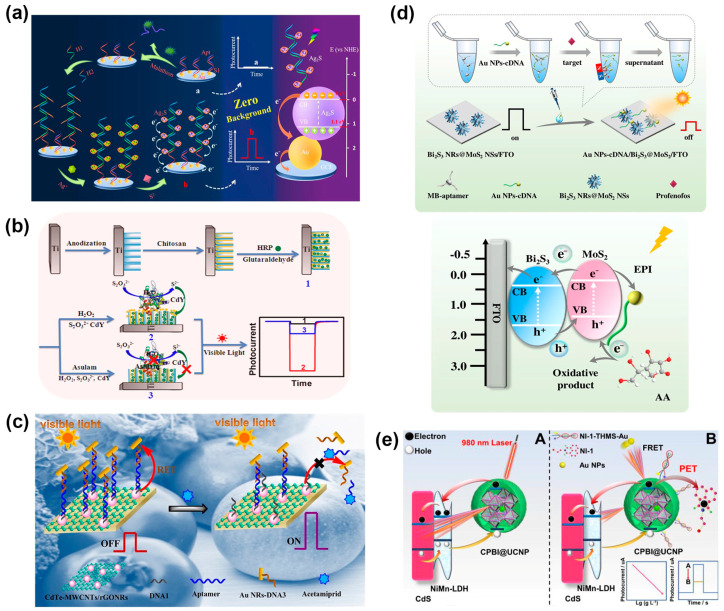
(**a**) Schematic illustration of the PEC sensor based on HCR and in situ generation of Ag_2_S photosensitive material for the detection of malathion [151]. Copyright 2021 Springer Nature. (**b**) Schematic illustration of the construction of a PEC sensor for asulam detection based on the in situ synthesis of CdS QDs [153]. Copyright 2018 Elsevier. (**c**) Schematic illustration of the PEC acetamiprid aptasensor based on the RET from CdTe QDs to Au NRs [156]. Copyright 2016 Elsevier. (**d**) Schematic illustration of the split-type PEC sensor for profenofos detection based on magnetic-assisted exciton–plasmon interactions [157]. Copyright 2023 Springer Nature. (**e**) Schematic illustration of the PEC sensor based on the cascade sensitization effect and synergetic quenching effect of FRET/PET [158]. Copyright 2023 American Chemical Society.

Zeng and coworkers reported a signal-on PEC sensor to determine malathion based on the in situ formation of DNA-templated Ag_2_S photoactive materials [151] (Figure 6a). The target-induced hybridization chain reaction (HCR) caused the exposure of rich “C” strands to assemble multiple silver ions by C-Ag^+^-C chelation. Afterwards, the Ag_2_S was generated in situ through the reaction between S^2−^ and Ag^+^ ions, presenting a desirable PEC signal. This signaling strategy dispensed with the pre-synthesis or immobilization of the photoactive materials, so it had nearly zero background noise, which was desirable for a high sensitivity with an LOD of 2 pg/mL. Meanwhile, the PEC sensor was also successfully applied to detect malathion in real samples. In another work, a target (malathion)-induced HCR can produce a long DNA concatemer containing the sequence of the G-quadruplex structure, then obtain HRP-like activity via the formation of hemin/G-quadruplex complexes and binding with manganese porphyrin [152]. With the aid of H_2_O_2_, the HRP-mimicking DNA concatemer catalyzed S_2_O_3_^2−^ to generate S^2−^, which could react with BiOBr nanoflowers to in situ form a BiOBr/Bi_2_S_3_ heterostructures with enhanced PEC conversion efficiency. The proposed PEC sensor achieved a wide linear range of 0.001–1000 ng/mL and an LOD of 0.12 pg/mL for malathion determination, and its application potential was verified by using it for the detection of spiked milk samples.

Inhibiting the generation of photoactive materials or releasing photoactive materials from the photoelectrode will result in a drop in the PEC signal. For example, a PEC sensor for asulam detection was developed based on the inhibitory effect on HRP enzyme activity and the lower production of S^2−^ from the conversion of S_2_O_3_^2−^ [153] (Figure 6b). Consequently, the amount of in situ-generated CdS reduced, and the photocurrent decreased with the concentration of asulam in a range from 0.02 to 2.0 ng/mL. The PEC sensor showed an LOD of 4.1 pg/mL and good accuracy in the analysis of spiked environmental water samples. Qin and coworkers proposed a PEC sensing platform for paraoxon detection by using a dissociable photoelectrode based on CdS nanocrystal-functionalized MnO_2_ NSs [154]. TCh can effectively etch MnO_2_ NSs, causing the dissociation of MnO_2_-CdS from the photoelectrode and the decline of the photocurrent response. With the presence of organophosphate pesticides, the generation of TCh and the dissociation of MnO_2_ NSs were prevented due to the inhibition of AChE, thus recovering the PEC signals. Upon this signaling strategy, rolling circle amplification was introduced to assemble a large number of butyrylcholinesterase (BChE) for increased production of TCh and promoted dissociation of MnO_2_-CdS [155]. Based on the inhibition of BChE by malathion, the PEC sensor possessed high sensitivity for malathion with a low LOD of 0.68 pg/mL and favorable accuracy in the analysis of spiked red wine and milk samples.

### 4.5. Energy Transfer

Energy transfer between the photoactive materials (namely, energy donor and acceptor) is a powerful approach to modulating the PEC properties of the PEC sensor. For PEC sensing of pesticides, the energy transfer strategy can be designed based on the recognition events, and the induced varying charge transfer behaviors will change the PEC conversion efficiency of the photoactive materials as well as the PEC signal [37].

Liu and coworkers reported a resonance energy transfer (RET) system from CdTe QDs to Au nanorods (NRs) for PEC aptasensing of acetamiprid [156]. Multi-walled carbon nanotubes/reduced graphene oxide nanoribbons (MWCNTs/rGONRs) served as the PEC signal amplifier and support for CdTe QDs loading, and the proposed CdTe-MWCNTs/rGONRs exhibited the typical fluorescence emission overlapped with the UV-vis absorption spectrum of Au NRs, leading to a RET process and photocurrent decrease. The binding of acetamiprid with aptamer resulted in the disassembly of the aptamer and low loading of Au NRs at the photoelectrode surface, thus inhibiting the RET process and recovering the photocurrent response (Figure 6c). The proposed PEC aptasensor was able to detect acetamiprid concentrations in the range of 0.5 pM to 10 μM with an LOD of 0.2 pM and was successfully applied to the direct detection of spiked apple and tomato samples. A split-type PEC sensor was designed for the detection of profenofos based on magnetic-assisted exciton–plasmon interactions (EPIs) [157] (Figure 6d). The target-triggered DNA double-stranded structure opening event released Au NPs-cDNA, which had significant overlap in the spectra of the Bi_2_S_3_/MoS_2_ substrate. The simultaneous excitation of both excitons and plasmons led to EPI between Bi_2_S_3_/MoS_2_ and Au NPs induced by SPR, which effectively suppressed the photocurrent generation. The PEC sensor exhibited good performance with a wide linear range (1.0 pg/mL–1.0 μg/mL), a low LOD (0.23 pg/mL), and satisfactory results in the analysis of spiked milk, cucumber, wastewater, and soil samples. Wang and coworkers designed a perovskite/upconversion lab-on-paper PEC device for the sensitive detection of malathion based on CsPbBr_2_I@NaYF_4_:Yb,Tm/NiMn-layered double hydroxide/CdS/Au NPs functionalized working electrode and entropy-driven strand displacement amplification with a triple helix molecules switch strategy [158] (Figure 6e). A synergistic quenching effect including fluorescence energy resonance transfer (FRET) and photoinduced electron transfer (PET) was customized to efficiently decrease the photocurrent, achieving sensitive detection of malathion with a linear range from 0.01 ng/L to 5 μg/L and an LOD of 4.8 fg/L. The applicability of the PEC device was verified by determining malathion in spiked water, cabbage juice, spinach juice, and soil samples.

A summary of the different categories of photoactive materials and signaling mechanisms-based PEC sensors for pesticide sensing in food and environmental samples is recorded in Table 1. Combining various advanced photoactive materials with well-designed recognition and signaling strategies, some of the reported PEC sensors can detect pesticides with a wide linear range of several orders of magnitude and high sensitivity down to the picomole scale. The applicabilities of these PEC sensing platforms have been demonstrated by real sample analysis of food and environmental samples, such as vegetables, fruits, beverages, water, and soil samples. Meanwhile, the employed light source is necessary and important for the PEC conversion process. It can be seen that most of the PEC sensing systems utilized xenon (Xe) lamps, halogen lamps, and light-emitting diode (LED) energy-saving lamps as light sources to simulate natural sunlight, and some of the light sources were equipped with monochromators or UV cut-off filters to achieve visible-light excitation and avoid the harmful effects caused by UV irradiation. The applied bias provides driving forces for charge transfer from photoactive materials to photoelectrodes. Most of the PEC sensors in Table 1 used relatively low applied biases, for example, around 0 V. Compared to traditional electrochemical methods, PEC sensing methods are less dependent on the applied potential, which is not only energy-saving but also beneficial for a lower background and higher sensitivity.

## 5. Conclusions and Perspectives

PEC sensing has emerged as a promising method for the detection of pesticide residue levels in food and environmental samples. Its advantages, such as high sensitivity and selectivity, portability, rapid analysis, and user-friendly operation, make it a futuristic option for pesticide analysis. In recent years, there has been a significant development of novel PEC sensing systems and new sensing protocols for pesticide determination. This review provides a comprehensive summary of the progress in the development of PEC sensors for pesticide analysis, focusing on PEC sensing principles, categories of photoactive materials, PEC signaling mechanisms, and their applications in food and environmental media analysis. The integration of photoactive materials with high PEC conversion efficiency and signaling mechanisms with wise transduction strategies has significantly enhanced the sensitivity, selectivity, and application performance of PEC sensors.

In spite of advances, significant work still remains in the research on PEC sensing of pesticides. On one hand, the PEC sensing method still has some common limitations: (1) The inherent background signal of the detection method remains challenging for current PEC sensors. Self-referencing PEC sensors based on different voltages or wavelengths with dual channels have been proposed to detect several types of targets, and additional efforts should be put into constructing ratiometric PEC sensors for pesticide analysis with high sensitivity. (2) Most of the PEC sensing systems contain lamps as light sources, bulky photo-to-electrochemical signal conversion equipment, and external power sources. To meet the requirements of on-site pesticide analysis, utilizing sunlight as a light source, replacing conventional electrochemical workstations with miniaturized signaling devices, and using screen-printing electrodes or transparent paper-based conductive electrodes with high light transmittance as working electrodes may improve portability and practicality. (3) Almost all reported PEC sensors are just carried out in laboratory research. Future efforts should be put into combining electronic information, micro-nanofabrication, and mass manufacturing to develop integrated detection devices with practicability. 

On the other hand, as to the PEC sensing of pesticides, the following aspects should be considered besides the above limitations: Firstly, the coexistence of multiple toxic elements has led to a notable increase in threats to food and environmental safety. Most of the currently developed PEC sensing systems focus on only one pesticide and are not able to meet current needs. The construction of a PEC sensing platform that simultaneously detects multiple pesticides can better satisfy real needs. Secondly, the applicability of proposed PEC sensors for pesticide detection is mostly verified through the analysis of spiked samples or spiked sample extracts at present, and the application of real-life samples is still rare. Overall, applying the developed PEC sensors on the user end is the final goal in this field. As the aforementioned requirements are continuously met, the PEC sensing technique is poised to play a pivotal role in pesticide detection for food and environmental safety.

## Figures and Tables

**Table 1:** A summary of the different categories of photoactive materials and signaling mechanisms-based PEC sensors for pesticide sensing in food and environmental samples.

Photoactive Materials	Light Sources	Applied Biases	Recognition Elements	Signaling Mechanisms	Target Pesticides	Detection Ranges	LODs	Real Samples	References
TiO_2−x_ NR/NGH	Xe lamp (350 W, 100 mW/cm^2^)	-	-	oxidation of pesticides	chlorpyrifos	0.05 ng/mL–0.5 μg/mL	0.017 ng/mL	wastewater	[122]
TiO_2_/P3HT/IL	halogen lamp (250 W)	0.2 V	-	oxidation of pesticides	acetochlor	0.5–20 μM	0.2 nM	water	[97]
PoPD@TiO_2_ NTs	Xe lamp (500 W, 460 nm)	0 V	MIP	oxidation of pesticides	lindane	0.1–10 μM	0.03 μM	drinking water, river water	[119]
PoPD-AuNPs/TiO_2_ NTs	Xe lamp (420 nm, 20 mW/cm^2^)	0 V	MIP	oxidation of pesticides	chlorpyrifos	0.05–10 μM	0.96 nM	green vegetable	[121]
PDPB-BiOBr	Xe lamp (300 W, with a 420 nm filter)	−0.2 V	aptamer	steric hindrance	acetamiprid	0.1 pM–10 μM	0.05 pM	cucumber, apple	[127]
TNA/g-C_3_N_4_	Xe lamp (500 W, with a 420 nm UV filter)	0.2 V	aptamer	steric hindrance	acetamiprid	0.1 pM–1.0 nM	0.025 pM	tomato, cucumber, bitter gourd	[92]
S-BN/Au/CN	Xe lamp (300 W)	0 V	aptamer	steric hindrance	diazinon	0.01–10,000 nM	6.8 pM	river water, tap water, apple	[128]
MoTe_2_ NPs/rGO	Xe lamp (250 W, λ > 400 nm)	0 V	aptamer	steric hindrance	profenofos	10^−9^–10^−2^ g/L	3.3 × 10^−10^ g/L	Chinese chive, potato	[99]
Pd NPs/CdS	Xe lamp (250 W)	0 V	aptamer	steric hindrance	carbendazim	1.0 × 10^−12^–1.0 × 10^−6^ M	3.3 × 10^−13^ M	lettuce	[113]
ZnO/Bi_2_O_3_/Bi_2_S_3_	Xe lamp (300 W, λ > 420 nm)	0 V	MIP	steric hindrance	thiamethoxam	7.0 × 10^−13^–7.0 × 10^−10^ M	3.32 × 10^−13^ M	lake water, soil leaching solutions	[131]
AgBiS_2_/Bi_2_S_3_	Xe lamp	0 V	MIP	steric hindrance	propoxur	1.0 × 10^−12^–5.0 × 10^−10^ M	2.3 × 10^−13^ M	pear, loquat, plum	[132]
B-TiO_2_ NRs	365 nm UV light	0 V	MIP	steric hindrance	chlorpyrifos	0.01–100 ng/mL	7.4 pg/mL	drinking water, river water	[133]
Sn_3_O_4_@CFP	WLC02 (500 W, 564 ± 60 nm)	0 V	MIP	steric hindrance	2,4-D	5.0 × 10^−11^–1.0 × 10^−7^ M	1.08 × 10^−11^ M	bean sprouts	[134]
I-BiOCl/N-GQD	Xe lamp (500 W, λ > 400 nm)	−0.1 V	BiOCl	steric hindrance	chlorpyrifos	0.3–80 ng/mL	0.01 ng/mL	river water	[139]
BiOI/TiO_2_	Xe lamp (300 W)	0 V	BiOI	steric hindrance	chlorpyrifos	1 pg/mL–200 ng/mL	0.24 pg/mL	tap water, lake water, lettuce, pitaya	[93]
Cu-BTC/CN-NS	LED energy-saving lamps (9 W)	0 V	Cu-BTC	steric hindrance	glyphosate	1.0 × 10^−12^–1.0 × 10^−8^ M, 1.0 × 10^−8^–1.0 × 10^−3^ M	1.3 × 10^−13^ M	soybean	[135]
CuO	LED energy-saving lamps (9 W)	0 V	CuO	steric hindrance	malathion	1.0 × 10^−10^–1.0 × 10^−5^ M	8.6 × 10^−11^ M	Chinese cabbage	[88]
Co_9_S_8_@CdS	Xe lamp (300 W, with 420 nm filter)	-	Co_9_S_8_	steric hindrance	chlorpyrifos	0.050–1000 ppb	0.015 ppb	wastewater	[140]
PTCA/TiO_2_	tungsten halogen lamp (250 W)	0.2 V	-	Generation/decrease in sacrificial agents	parathion-methyl	0.1–10 nM	0.08 nM	green vegetable	[144]
CdCoS_2_(2)@Ag	Xe lamp (420 nm, 20 mW/cm^2^)	0 V	AChE	Generation/decrease in sacrificial agents	chlorpyrifos	0.001–270 μg/mL	0.57 ng/mL	river water	[115]
ZnO/GAs	Xe lamp (250 W, with 400 nm UV filter, 100 mW/cm^2^)	0 V	AChE	Generation/decrease in sacrificial agents	parathion-methyl	0.1 ng/mL–0.1 μg/mL	0.03 ng/mL	cucumber, juice	[146]
NCQD/TiO_2_	Xe lamp (350 W, λ ≥ 420 nm, 20 mW/cm^2^)	0 V	AChE	Generation/decrease in sacrificial agents	chlorpyrifos	0.001–1.5 μg/mL	0.07 ng/mL	lake water, Chinese cabbage	[147]
Bi_2_S_3_@Bi_2_Sn_2_O_7_	Xe lamp (500 W)	0.2 V	ALP	Generation/decrease in sacrificial agents	omethoate	0.05–500 ng/mL	0.0146 ng/mL	spinach, mustard	[149]
NH_2_-MIL-125(Ti)/TiO_2_	halogen lamp (250 W, λ > 400 nm)	0.2 V	GOx	Generation/decrease in sacrificial agents	acetochlor	0.02–1.0 nM, 10–200 nM	0.003 nM	strawberry, tomato, cucumber, greens	[150]
Au NPs/Ag_2_S	Xe lamp (500 W)	1.0 V	aptamer	Introduction/release of photoactive materials	malathion	0.006–600 ng/mL	2 pg/mL	apple juice	[151]
BiOBr/Bi_2_S_3_	Xe lamp (500 W, with 420 nm filter)	-	aptamer	Introduction/release of photoactive materials	malathion	0.001–1000 ng/mL	0.12 pg/mL	milk	[152]
TNA/CdS	Xe lamp (500 W, with a 400 nm UV filter)	0.2 V	HRP	Introduction/release of photoactive materials	asulam	0.02–2.0 ng/mL	4.1 pg/mL	lake water, river water, drinking water	[153]
MnO_2_-CdS	Xe lamp (500 W, with 420 nm UV filter)	−0.3 V	AChE	Introduction/release of photoactive materials	paraoxon	0.05–10 ng/mL	0.017 ng/mL	tap water	[154]
MnO_2_ NF@CdS	Xe lamp (500 W)	0.01 V	BchE	Introduction/release of photoactive materials	malathion	0.001–100 ng/mL	0.68 pg/mL	red wine, milk	[155]
CdTe-MWCNTs/rGONRs-Au NRs	Xe lamp (250 W, with 400 nm UV filter, 100 mW/cm^2^)	0.1 V	aptamer	Energy transfer	acetamiprid	0.5 pM–10 μM	0.2 pM	apple, tomato	[156]
Bi_2_S_3_ NRs @MoS_2_ NSs-Au NPs	LED (5 W, 450 nm)	0 V	aptamer	Energy transfer	profenofos	1.0 pg/mL–1.0 μg/mL	0.23 pg/mL	milk, cucumber, wastewater, soil	[157]
CPBI@UCNP/NiMn-LDH/CdS-Au NPs-NI-1	980 nm NIR laser (1.5 W/cm^2^)	-	aptamer	Energy transfer	malathion	0.01 ng/L–5 μg/L	4.8 fg/L	water, cabbage juice, spinach juice, soil	[158]

## Data Availability

The authors declare that all data generated or analyzed during this study are included in the published article.

## References

[1] Rosenthal T. (2007). Pesticides: Health, Safety, and the Environment. J. Rural Health.

[2] Nsibande S.A., Forbes P.B.C. (2016). Fluorescence Detection of Pesticides Using Quantum Dot Materials—A Review. Anal. Chim. Acta.

[3] Zamora-Sequeira R., Starbird-Pérez R., Rojas-Carillo O., Vargas-Villalobos S. (2019). What Are the Main Sensor Methods for Quantifying Pesticides in Agricultural Activities? A Review. Molecules.

[4] Bose S., Kumar P.S., Vo D.-V.N., Rajamohan N., Saravanan R. (2021). Microbial Degradation of Recalcitrant Pesticides: A Review. Environ. Chem. Lett..

[5] Gonçalves-Filho D., Silva C.C.G., De Souza D. (2020). Pesticides Determination in Foods and Natural Waters Using Solid Amalgam-Based Electrodes: Challenges and Trends. Talanta.

[6] Bini Dhouib I., Annabi A., Jallouli M., Marzouki S., Gharbi N., Elfazaa S., Montassar Lasram M. (2016). Carbamates Pesticides Induced Immunotoxicity and Carcinogenicity in Human: A Review. J. Appl. Biomed..

[7] Hui T., Xiujuan L., Qifa S., Qiang L., Zhuang K., Yan G. (2020). Evaluation of Drinking Water Quality Usingthe Water Quality Index (WQI), the SyntheticPollution Index (SPI) and Geospatial Toolsin Lianhuashan District, China. Pol. J. Environ. Stud..

[8] Carvalho F.P. (2017). Pesticides, Environment, and Food Safety. Food Energy Secur..

[9] Udeigwe T.K., Teboh J.M., Eze P.N., Hashem Stietiya M., Kumar V., Hendrix J., Mascagni H.J., Ying T., Kandakji T. (2015). Implications of Leading Crop Production Practices on Environmental Quality and Human Health. J. Environ. Manag..

[10] Boedeker W., Watts M., Clausing P., Marquez E. (2020). The Global Distribution of Acute Unintentional Pesticide Poisoning: Estimations Based on a Systematic Review. BMC Public Health.

[11] Thundiyil J. (2008). Acute Pesticide Poisoning: A Proposed Classification Tool. Bull. World Health Organ..

[12] Mostafalou S., Abdollahi M. (2017). Pesticides: An Update of Human Exposure and Toxicity. Arch. Toxicol..

[13] Beseler C.L., Stallones L., Hoppin J.A., Alavanja M.C.R., Blair A., Keefe T., Kamel F. (2008). Depression and Pesticide Exposures among Private Pesticide Applicators Enrolled in the Agricultural Health Study. Environ. Health Perspect..

[14] Eddleston M. (2020). Poisoning by Pesticides. Medicine.

[15] The Innovation Editorial Team, China (2022). Editorial Innovation Focus in 2021. Innovation.

[16] Zikankuba V.L., Mwanyika G., Ntwenya J.E., James A. (2019). Pesticide Regulations and Their Malpractice Implications on Food and Environment Safety. Cogent Food Agric..

[17] Handford C.E., Elliott C.T., Campbell K. (2015). A Review of the Global Pesticide Legislation and the Scale of Challenge in Reaching the Global Harmonization of Food Safety Standards. Integr. Environ. Assess. Manag..

[18] Zhang Y., Si W., Chen L., Shen G., Bai B., Zhou C. (2021). Determination and Dietary Risk Assessment of 284 Pesticide Residues in Local Fruit Cultivars in Shanghai, China. Sci. Rep..

[19] Jiang W., Li Z., Yang Q., Hou X. (2023). Integration of Metallic Nanomaterials and Recognition Elements for the Specifically Monitoring of Pesticides in Electrochemical Sensing. Crit. Rev. Anal. Chem..

[20] Li J., Wang Z., Li J., Zhang S., An Y., Hao L., Yang X., Wang C., Wang Z., Wu Q. (2022). Novel N-Riched Covalent Organic Framework for Solid-Phase Microextraction of Organochlorine Pesticides in Vegetable and Fruit Samples. Food Chem..

[21] Senosy I.A., Lu Z.-H., Zhou D.-D., Abdelrahman T.M., Chen M., Zhuang L.-Y., Liu X., Cao Y.-W., Li J.-H., Yang Z.H. (2022). Construction of a Magnetic Solid-Phase Extraction Method for the Analysis of Azole Pesticides Residue in Medicinal Plants. Food Chem..

[22] Osaili T.M., Al-Natour M.Q., Al-Abboodi A.R., Alkarasneh A.Y., El Darra N., Khazaal S., Holley R. (2023). Detection and Risk Associated with Organochlorine, Organophosphorus, Pyrethroid and Carbamate Pesticide Residues in Chicken Muscle and Organ Meats in Jordan. Food Control..

[23] Yang Y., Zheng K., Guo L.-P., Wang C.-X., Zhong D.-B., Shang L., Nian H.-J., Cui X.-M., Huang S.-J. (2022). Rapid Determination and Dietary Intake Risk Assessment of 249 Pesticide Residues in Panax Notoginseng. Ecotoxicol. Environ. Saf..

[24] Samsidar A., Siddiquee S., Shaarani S.M. (2018). A Review of Extraction, Analytical and Advanced Methods for Determination of Pesticides in Environment and Foodstuffs. Trends Food Sci. Technol..

[25] Ben Attig J., Latrous L., Zougagh M., Ríos Á. (2021). Ionic Liquid and Magnetic Multiwalled Carbon Nanotubes for Extraction of N-Methylcarbamate Pesticides from Water Samples Prior Their Determination by Capillary Electrophoresis. Talanta.

[26] Jiang M., Chen C., He J., Zhang H., Xu Z. (2020). Fluorescence Assay for Three Organophosphorus Pesticides in Agricultural Products Based on Magnetic-Assisted Fluorescence Labeling Aptamer Probe. Food Chem..

[27] Chen Z., Zhao L., Zhang Z., Wu J., Zhang L., Jing X., Wang X. (2023). Dispersive Liquid–liquid Microextraction Combined with Enzyme-Linked Immunosorbent Assay for the Analysis of Chlorpyrifos in Cereal Samples. Talanta.

[28] Ma L., Xu Q., Yin L., Wu W., Han E., Wang C., Zhou R., Bai J., Cai J. (2024). Simultaneous Detection of Acetamiprid and Carbendazim Based on Raman-Silent Spectral Window Tags-Mediated Surface-Enhanced Raman Scattering Aptasensor Coupled with Magnetic Separation. Sens. Actuators B Chem..

[29] Li H., Sheng W., Haruna S.A., Bei Q., Wei W., Hassan M.M., Chen Q. (2023). Recent Progress in Photoelectrochemical Sensors to Quantify Pesticides in Foods: Theory, Photoactive Substrate Selection, Recognition Elements and Applications. TrAC Trends Anal. Chem..

[30] Mishra A., Kumar J., Melo J.S., Sandaka B.P. (2021). Progressive Development in Biosensors for Detection of Dichlorvos Pesticide: A Review. J. Environ. Chem. Eng..

[31] Ding R., Li Z., Xiong Y., Wu W., Yang Q., Hou X. (2023). Electrochemical (Bio)Sensors for the Detection of Organophosphorus Pesticides Based on Nanomaterial-Modified Electrodes: A Review. Crit. Rev. Anal. Chem..

[32] Zhao W.-W., Xu J.-J., Chen H.-Y. (2015). Photoelectrochemical Bioanalysis: The State of the Art. Chem. Soc. Rev..

[33] Qureshi A., Shaikh T., Niazi J.H. (2023). Semiconductor Quantum Dots in Photoelectrochemical Sensors from Fabrication to Biosensing Applications. Analyst.

[34] Shi J., Chen Z., Zhao C., Shen M., Li H., Zhang S., Zhang Z. (2022). Photoelectrochemical Biosensing Platforms for Tumor Marker Detection. Coord. Chem. Rev..

[35] Zang Y., Fan J., Ju Y., Xue H., Pang H. (2018). Current Advances in Semiconductor Nanomaterial-Based Photoelectrochemical Biosensing. Chem. Eur. J..

[36] Zhou Q., Tang D. (2020). Recent Advances in Photoelectrochemical Biosensors for Analysis of Mycotoxins in Food. TrAC Trends Anal. Chem..

[37] Qiu Z., Tang D. (2020). Nanostructure-Based Photoelectrochemical Sensing Platforms for Biomedical Applications. J. Mater. Chem. B.

[38] Zhao C.-Q., Ding S.-N. (2019). Perspective on Signal Amplification Strategies and Sensing Protocols in Photoelectrochemical Immunoassay. Coord. Chem. Rev..

[39] Liu Y., Li R., Gao P., Zhang Y., Ma H., Yang J., Du B., Wei Q. (2015). A Signal-off Sandwich Photoelectrochemical Immunosensor Using TiO_2_ Coupled with CdS as the Photoactive Matrix and Copper (II) Ion as Inhibitor. Biosens. Bioelectron..

[40] Bilge S., Sınağ A. (2023). Current Trends and Strategies in the Development of Green MXene-Based Photoelectrochemical Sensing Application. TrAC Trends Anal. Chem..

[41] Ge L., Liu Q., Hao N., Kun W. (2019). Recent Developments of Photoelectrochemical Biosensors for Food Analysis. J. Mater. Chem. B.

[42] Shu J., Tang D. (2020). Recent Advances in Photoelectrochemical Sensing: From Engineered Photoactive Materials to Sensing Devices and Detection Modes. Anal. Chem..

[43] Zhao D., Geng C., Liu X., Jin X., Zhao Z., Liu Y., Alwarappan S. (2023). Photoelectrochemical Detection of Superoxide Anions Released from Mitochondria in HepG2 Cells Based on the Synergistic Effect of MnO_2_@Co_3_O_4_ Core-Shell p-n Heterojunction. Biosens. Bioelectron..

[44] Xiao K., Zhu R., Du C., Zhang X., Chen J. (2023). Highly Sensitive and Selective microRNA Photoelectrochemical Assay with Magnetic Electron Donor–Acceptor Covalent Organic Framework as Photoactive Material and ZnSe QDs as Photocurrent-Polarity-Switching Factor. Sens. Actuators B Chem..

[45] Li H., Wang J., Wang X., Lin H., Li F. (2019). Perylene-Based Photoactive Material as a Double-Stranded DNA Intercalating Probe for Ultrasensitive Photoelectrochemical Biosensing. ACS Appl. Mater. Interfaces.

[46] Tao B., Yang W., Miao F., Zang Y. (2023). Co_3_O_4_/ZnO/CNTs Photoelectrochemical Sensor for Sensitive and Selective LA Detection. IEEE Trans. Electron Devices.

[47] Yang Z.-Y., Song J., Cai T.-X., Jiang R.-L., Dai J., Xu K., Yang X.-L., Xie M.-H. (2023). Construction of TiO_2_@ZnO Nanofibers with Beads-on-a-String Heterostructures for Photoelectrochemical Detection of Lactic Acid. J. Alloys Compd..

[48] Yan T., Zhang G., Yu K., Chai H., Tian M., Qu L., Dong H., Zhang X. (2023). Smartphone Light-Driven Zinc Porphyrinic MOF Nanosheets-Based Enzyme-Free Wearable Photoelectrochemical Sensor for Continuous Sweat Vitamin C Detection. Chem. Eng. J..

[49] Neven L., Shanmugam S.T., Rahemi V., Trashin S., Sleegers N., Carrión E.N., Gorun S.M., De Wael K. (2019). Optimized Photoelectrochemical Detection of Essential Drugs Bearing Phenolic Groups. Anal. Chem..

[50] Jacques R., Zhou B., Marhuenda E., Gorecki J., Das A., Iskratsch T., Krause S. (2023). Photoelectrochemical Imaging of Single Cardiomyocytes and Monitoring of Their Action Potentials through Contact Force Manipulation of Organoids. Biosens. Bioelectron..

[51] Chen X., Wu W., Zeng J., Ibañez E., Cifuentes A., Mao J., Yu L., Wu H., Li P., Zhang Z. (2023). A Smartphone-Powered Photoelectrochemical POCT via Z-Scheme Cu_2_O/Cu_3_SnS_4_ for Dibutyl Phthalate in the Environmental and Food. J. Hazard. Mater..

[52] Dai H., Yin M., Zhang S., Wei J., Jiao T., Chen Q., Chen Q., Chen X., Oyama M., Chen X. (2024). A Paper-Based Photoelectrochemical Aptsensor Using near-Infrared Light-Responsive AgBiS_2_ Nanoflowers as Probes for the Detection of Staphylococcus Aureus in Pork. Talanta.

[53] Ma X., Kang J., Wu Y., Pang C., Li S., Li J., Xiong Y., Luo J., Wang M., Xu Z. (2022). Recent Advances in Metal/Covalent Organic Framework-Based Materials for Photoelectrochemical Sensing Applications. TrAC Trends Anal. Chem..

[54] Yang L., Zhang S., Liu X., Tang Y., Zhou Y., Wong D.K.Y. (2020). Detection Signal Amplification Strategies at Nanomaterial-Based Photoelectrochemical Biosensors. J. Mater. Chem. B.

[55] Zang Y., Lei J., Ju H. (2017). Principles and Applications of Photoelectrochemical Sensing Strategies Based on Biofunctionalized Nanostructures. Biosens. Bioelectron..

[56] Tu W., Wang Z., Dai Z. (2018). Selective Photoelectrochemical Architectures for Biosensing: Design, Mechanism and Responsibility. TrAC Trends Anal. Chem..

[57] Jia M., Xiong W., Yang Z., Cao J., Zhang Y., Xiang Y., Xu H., Song P., Xu Z. (2021). Metal-Organic Frameworks and Their Derivatives-Modified Photoelectrodes for Photoelectrochemical Applications. Coord. Chem. Rev..

[58] Zhou Y., Yin H., Ai S. (2023). Recent Advances and Applications of Bi_2_S_3_-Based Composites in Photoelectrochemical Sensors and Biosensors. TrAC Trends Anal. Chem..

[59] Liu Q., Huan J., Hao N., Qian J., Mao H., Wang K. (2017). Engineering of Heterojunction-Mediated Biointerface for Photoelectrochemical Aptasensing: Case of Direct Z-Scheme CdTe-Bi_2_S_3_ Heterojunction with Improved Visible-Light-Driven Photoelectrical Conversion Efficiency. ACS Appl. Mater. Interfaces.

[60] You F., Zhu M., Ding L., Xu Y., Wang K. (2019). Design and Construction of Z-Scheme Bi_2_S_3_/Nitrogen-Doped Graphene Quantum Dots: Boosted Photoelectric Conversion Efficiency for High-Performance Photoelectrochemical Aptasensing of Sulfadimethoxine. Biosens. Bioelectron..

[61] Yin Z., Zhi J. (2020). A Photoelectrochemical Biosensor Based on the Direct Electron Transfer to Galactose Oxidase. J. Photochem. Photobiol. Chem..

[62] Xia L., Song J., Xu R., Liu D., Dong B., Xu L., Song H. (2014). Zinc Oxide Inverse Opal Electrodes Modified by Glucose Oxidase for Electrochemical and Photoelectrochemical Biosensor. Biosens. Bioelectron..

[63] Du J., Yu X., Wu Y., Di J. (2013). ZnS Nanoparticles Electrodeposited onto ITO Electrode as a Platform for Fabrication of Enzyme-Based Biosensors of Glucose. Mater. Sci. Eng. C.

[64] Wang H., Wan X., Wang X., Li M., Tang D. (2023). Ultrathin Mesoporous BiOCl Nanosheets-Mediated Liposomes for Photoelectrochemical Immunoassay with In-Situ Signal Amplification. Biosens. Bioelectron..

[65] Ma Z.-Y., Pan J.-B., Lu C.-Y., Zhao W.-W., Xu J.-J., Chen H.-Y. (2014). Folding-Based Photoelectrochemical Biosensor: Binding-Induced Conformation Change of a Quantum Dot-Tagged DNA Probe for Mercury(II) Detection. Chem. Commun..

[66] Wang W., Bao L., Lei J., Tu W., Ju H. (2012). Visible Light Induced Photoelectrochemical Biosensing Based on Oxygen-Sensitive Quantum Dots. Anal. Chim. Acta.

[67] Wang G.-L., Liu K.-L., Shu J.-X., Gu T.-T., Wu X.-M., Dong Y.-M., Li Z.-J. (2015). A Novel Photoelectrochemical Sensor Based on Photocathode of PbS Quantum Dots Utilizing Catalase Mimetics of Bio-Bar-Coded Platinum Nanoparticles/G-Quadruplex/Hemin for Signal Amplification. Biosens. Bioelectron..

[68] Wang M., Yang Z., Guo Y., Wang X., Yin H., Ai S. (2015). Visible-Light Induced Photoelectrochemical Biosensor for the Detection of microRNA Based on Bi_2_S_3_ Nanorods and Streptavidin on an ITO Electrode. Microchim. Acta.

[69] Chen L., Chen Y., Miao L., Gao Y., Di J. (2020). Photocurrent Switching Effect on BiVO_4_ Electrodes and Its Application in Development of Photoelectrochemical Glucose Sensor. J. Solid State Electrochem..

[70] Dong X., Xu C., Yang C., Chen F., Manohari A.G., Zhu Z., Zhang W., Wang R., You D., Chen J. (2019). Photoelectrochemical Response to Glutathione in Au-Decorated ZnO Nanorod Array. J. Mater. Chem. C.

[71] Yang G., Chen X., Pan Q., Liu W., Zhao F. (2017). A Novel Photoelectrochemical Sensor for Thiamphenicol Based on Porous Three-Dimensional Imprinted Film. Int. J. Electrochem. Sci..

[72] Hu Y., Xue Z., He H., Ai R., Liu X., Lu X. (2013). Photoelectrochemical Sensing for Hydroquinone Based on Porphyrin-Functionalized Au Nanoparticles on Graphene. Biosens. Bioelectron..

[73] Wu S., Song H., Song J., He C., Ni J., Zhao Y., Wang X. (2014). Development of Triphenylamine Functional Dye for Selective Photoelectrochemical Sensing of Cysteine. Anal. Chem..

[74] Wu S., Tu W., Zhao Y., Wang X., Song J., Yang X. (2018). Phosphonate-Substituted Ruthenium(II) Bipyridyl Derivative as a Photoelectrochemical Probe for Sensitive and Selective Detection of Mercury(II) in Biofluids. Anal. Chem..

[75] Li Z., Dong W., Du X., Wen G., Fan X. (2020). A Novel Photoelectrochemical Sensor Based on G-C_3_N_4_@CdS QDs for Sensitive Detection of Hg^2+^. Microchem. J..

[76] Ye C., Wu Z., Ma K., Xia Z., Pan J., Wang M., Ye C. (2021). Ti_3_C_2_ MXene-Based Schottky Photocathode for Enhanced Photoelectrochemical Sensing. J. Alloys Compd..

[77] Kong W., Xiang M.-H., Xia L., Zhang M., Kong R.-M., Qu F. (2020). In-Situ Synthesis of 3D Cu_2_O@Cu-Based MOF Nanobelt Arrays with Improved Conductivity for Sensitive Photoelectrochemical Detection of Vascular Endothelial Growth Factor 165. Biosens. Bioelectron..

[78] Zhang X., Gao Y., Li J., Yan J., Liu P., Fan X., Song W. (2023). A Novel TAPP-DHTA COF Cathodic Photoelectrochemical Immunosensor Based on CRISPR/Cas12a-Induced Nanozyme Catalytic Generation of Heterojunction. Electrochim. Acta.

[79] Yu Y., Guo J., Zhang H., Wang X., Yang C., Zhao Y. (2022). Shear-Flow-Induced Graphene Coating Microfibers from Microfluidic Spinning. Innovation.

[80] Liu X., Verma G., Chen Z., Hu B., Huang Q., Yang H., Ma S., Wang X. (2022). Metal-Organic Framework Nanocrystal-Derived Hollow Porous Materials: Synthetic Strategies and Emerging Applications. Innovation.

[81] Pardo-Yissar V., Katz E., Wasserman J., Willner I. (2003). Acetylcholine Esterase-Labeled CdS Nanoparticles on Electrodes: Photoelectrochemical Sensing of the Enzyme Inhibitors. J. Am. Chem. Soc..

[82] Zhang X., Li S., Jin X., Li X. (2011). Aptamer Based Photoelectrochemical Cytosensor with Layer-by-Layer Assembly of CdSe Semiconductor Nanoparticles as Photoelectrochemically Active Species. Biosens. Bioelectron..

[83] Zhang B., Guo L.-H. (2012). Highly Sensitive and Selective Photoelectrochemical DNA Sensor for the Detection of Hg^2+^ in Aqueous Solutions. Biosens. Bioelectron..

[84] Ikeda A., Nakasu M., Ogasawara S., Nakanishi H., Nakamura M., Kikuchi J. (2009). Photoelectrochemical Sensor with Porphyrin-Deposited Electrodes for Determination of Nucleotides in Water. Org. Lett..

[85] Xu X., Zhou H., Zhang J., Li Y., Yang Y., Fang Y., Wu Z., Cui B., Hu Q. (2022). One-Step Electropolymerization of Polythiophene Derivative Film for Photoelectrochemical Detection of Chlorpyrifos. J. Electrochem. Soc..

[86] Gong J., Wang X., Li X., Wang K. (2012). Highly Sensitive Visible Light Activated Photoelectrochemical Biosensing of Organophosphate Pesticide Using Biofunctional Crossed Bismuth Oxyiodide Flake Arrays. Biosens. Bioelectron..

[87] Miao L., Li Z., Chen Y., Gao Y., Di J. (2023). A Sensitive Photoelectrochemical Biosensor for Pesticide Detection Based on BiVO_4_. Int. J. Environ. Anal. Chem..

[88] Cao Y., Wang L., Wang C., Su D., Liu Y., Hu X. (2019). Photoelectrochemical Determination of Malathion by Using CuO Modified with a Metal-Organic Framework of Type Cu-BTC. Microchim. Acta.

[89] Ma X., Wang C., Wu F., Guan Y., Xu G. (2020). TiO_2_ Nanomaterials in Photoelectrochemical and Electrochemiluminescent Biosensing. Top. Curr. Chem..

[90] Liu P.P., Liu X., Huo X.H., Tang Y., Xu J., Ju H. (2017). TiO_2_–BiVO_4_ Heterostructure to Enhance Photoelectrochemical Efficiency for Sensitive Aptasensing. ACS Appl. Mater. Interfaces.

[91] Fu B., Wu W., Gan L., Zhang Z. (2019). Bulk/Surface Defects Engineered TiO_2_ Nanotube Photonic Crystals Coupled with Plasmonic Gold Nanoparticles for Effective in Vivo Near-Infrared Light Photoelectrochemical Detection. Anal. Chem..

[92] Zhou C., Huang M., Huang W., Tian J., Zhang Y., Lu J. (2021). A Photoelectrochemical Aptasensor for Acetamiprid Determination Based on λ Exonuclease-Assisted Recycling Amplification and DNAzyme-Catalyzed Precipitation. J. Electrochem. Soc..

[93] Lyu R., Lei Y., Zhang C., Li G., Han R., Zou L. (2023). An Ultra-Sensitive Photoelectrochemical Sensor for Chlorpyrifos Detection Based on a Novel BiOI/TiO_2_ n-n Heterojunction. Anal. Chim. Acta.

[94] Lei Q., Li J., Lei J., Hong B., Li X., Li H., Wan Y. (2023). Photoelectrochemical Sensor Based on Bi_2_S_3_ @g-C_3_N_4_ Heterojunction for the Detection of Chlorpyrifos. Surf. Interfaces.

[95] Zhu Y., Dan Y. (2010). Photocatalytic Activity of Poly(3-Hexylthiophene)/Titanium Dioxide Composites for Degrading Methyl Orange. Sol. Energy Mater. Sol. Cells.

[96] Li H., Li J., Xu Q., Hu X. (2011). Poly(3-Hexylthiophene)/TiO_2_ Nanoparticle-Functionalized Electrodes for Visible Light and Low Potential Photoelectrochemical Sensing of Organophosphorus Pesticide Chlopyrifos. Anal. Chem..

[97] Jin D., Xu Q., Wang Y., Hu X. (2014). A Derivative Photoelectrochemical Sensing Platform for Herbicide Acetochlor Based on TiO_2_–Poly (3-Hexylthiophene)–Ionic Liquid Nanocomposite Film Modified Electrodes. Talanta.

[98] Song J., Wu S., Xing P., Zhao Y., Yuan J. (2018). Di-Branched Triphenylamine Dye Sensitized TiO_2_ Nanocomposites with Good Photo-Stability for Sensitive Photoelectrochemical Detection of Organophosphate Pesticides. Anal. Chim. Acta.

[99] Ding L., Wei J., Qiu Y., Wang Y., Wen Z., Qian J., Hao N., Ding C., Li Y., Wang K. (2021). One-Step Hydrothermal Synthesis of Telluride Molybdenum/Reduced Graphene Oxide with Schottky Barrier for Fabricating Label-Free Photoelectrochemical Profenofos Aptasensor. Chem. Eng. J..

[100] Yan Y., Li H., Liu Q., Hao N., Mao H., Wang K. (2017). A Facile Strategy to Construct Pure Thiophene-Sulfur-Doped Graphene/ZnO Nanoplates Sensitized Structure for Fabricating a Novel “on-off-on” Switch Photoelectrochemical Aptasensor. Sens. Actuators B Chem..

[101] Abd-Elrahim A.G., Chun D.-M. (2021). Facile One-Step Deposition of ZnO-Graphene Nanosheets Hybrid Photoanodes for Enhanced Photoelectrochemical Water Splitting. J. Alloys Compd..

[102] Kumar A.S., Reddy N.R., Sai K.N.S., Reddy G.R., Dhananjaya M., Kim J.S., Joo S.W. (2023). Design of P-CuS/n-CdFe_2_O_4_ Heterojunction Passivated with Carbon Nanofiber (CNF) for Impressive Performance in Supercapacitor and Photoelectrochemical Water Splitting. J. Alloys Compd..

[103] Umeyama T., Imahori H. (2008). Carbon Nanotube-Modified Electrodes for Solar Energy Conversion. Energy Environ. Sci..

[104] Qin Q., Bai X., Hua Z. (2017). Electrochemical Synthesis of Well-Dispersed CdTe Nanoparticles on Reduced Graphene Oxide and Its Photoelectrochemical Sensing of Catechol. J. Electrochem. Soc..

[105] Qian J., Yang Z., Wang C., Wang K., Liu Q., Jiang D., Yan Y., Wang K. (2015). One-Pot Synthesis of BiPO_4_ Functionalized Reduced Graphene Oxide with Enhanced Photoelectrochemical Performance for Selective and Sensitive Detection of Chlorpyrifos. J. Mater. Chem. A.

[106] Jiang D., Du X., Chen D., Li Y., Hao N., Qian J., Zhong H., You T., Wang K. (2016). Facile Wet Chemical Method for Fabricating P-Type BiOBr/n-Type Nitrogen Doped Graphene Composites: Efficient Visible-Excited Charge Separation, and High-Performance Photoelectrochemical Sensing. Carbon.

[107] Atabaev T.S., Hossain M.A., Lee D., Kim H.-K., Hwang Y.-H. (2016). Pt-Coated TiO_2_ Nanorods for Photoelectrochemical Water Splitting Applications. Results Phys..

[108] Wang D., Ding Z., Zhou H., Chen L., Feng X. (2021). Au Nanoparticle-Decorated TiO_2_ Nanowires for Surface Plasmon Resonance-Based Photoelectrochemical Bioassays with a Solid–Liquid–Air Triphase Interface. ACS Appl. Nano Mater..

[109] Zhang N., Li M., Tan C.F., Nuo Peh C.K., Sum T.C., Ho G.W. (2017). Plasmonic Enhanced Photoelectrochemical and Photocatalytic Performances of 1D Coaxial Ag@Ag_2_S Hybrids. J. Mater. Chem. A.

[110] Yu S.-Y., Zhang L., Zhu L.-B., Gao Y., Fan G.-C., Han D.-M., Chen G., Zhao W.-W. (2019). Bismuth-Containing Semiconductors for Photoelectrochemical Sensing and Biosensing. Coord. Chem. Rev..

[111] Deiminiat B., Rounaghi G.H. (2023). Fabrication of a Novel Photoelectrochemical Aptasensor Using Gold Nanoparticle-Sensitized TiO2 Film for Quantitative Determination of Diazinon in Solutions. Electrocatalysis.

[112] Zhang Z., Ding X., Lu G., Du B., Liu M. (2023). A Highly Sensitive and Selective Photoelectrochemical Aptasensor for Atrazine Based on Au NPs/3DOM TiO_2_ Photonic Crystal Electrode. J. Hazard. Mater..

[113] Wen Z., Zhu W., You F., Yuan R., Ding L., Hao N., Wei J., Wang K. (2022). Ultrasensitive Photoelectrochemical Aptasensor for Carbendazim Detection Based on In-Situ Constructing Schottky Junction via Photoreducing Pd Nanoparticles onto CdS Microsphere. Biosens. Bioelectron..

[114] Liu D., Gong Q., Xu X., Meng S., Li Y., You T. (2023). Photoelectrochemical Aptasensor Based on Cascade Dual Z-Scheme CdTe-polyaniline@MoS_2_ Heterostructure for the Sensitive Carbendazim Detection. J. Electroanal. Chem..

[115] Zheng D., Chen M., Chen Y., Gao W. (2022). In-Situ Preparation of Hollow CdCoS_2_ Heterojunction with Enhanced Photocurrent Response for Highly Photoelectrochemical Sensing of Organophosphorus Pesticides. Anal. Chim. Acta.

[116] Feng J., Li Y., Gao Z., Lv H., Zhang X., Fan D., Wei Q. (2018). Visible-Light Driven Label-Free Photoelectrochemical Immunosensor Based on TiO_2_/S-BiVO_4_@Ag_2_S Nanocomposites for Sensitive Detection OTA. Biosens. Bioelectron..

[117] Zhang C., Zhou L., Peng J. (2021). Blue-Light Photoelectrochemical Aptasensor for Kanamycin Based on Synergistic Strategy by Schottky Junction and Sensitization. Sens. Actuators B Chem..

[118] Zhou J., Liu Z., Ling Y., Zeng B., Zhang H., Deng K. (2022). Bismuth Oxyiodide-Bismuth Hybridized with Short Carbon Nanotubes as Cathode Photoelectrochemical Sensing Platform for Determination of Chlorpyrifos. Chin. J. Anal. Chem..

[119] Wang P., Ge L., Li M., Li W., Li L., Wang Y., Yu J. (2013). Photoelectrochemical Sensor Based on Molecularly Imprinted Polymer-Coated TiO_2_ Nanotubes for Lindane Specific Recognition and Detection. J. Inorg. Organomet. Polym. Mater..

[120] Wang H., Zhang B., Tang Y., Wang C., Zhao F., Zeng B. (2020). Recent Advances in Bismuth Oxyhalide-Based Functional Materials for Photoelectrochemical Sensing. TrAC Trends Anal. Chem..

[121] Wang P., Dai W., Ge L., Yan M., Ge S., Yu J. (2013). Visible Light Photoelectrochemical Sensor Based on Au Nanoparticles and Molecularly Imprinted Poly(o-Phenylenediamine)-Modified TiO_2_ Nanotubes for Specific and Sensitive Detection Chlorpyrifos. Analyst.

[122] Du X., Sun J., Jiang D., Du W. (2021). Non-Noble Metal Plasmonic Enhanced Photoelectrochemical Sensing of Chlorpyrifos Based on 1D TiO_2_-x/3D Nitrogen-Doped Graphene Hydrogel Heterostructure. Anal. Bioanal. Chem..

[123] Wen Z., Ding L., Zhu W., You F., Wang T., Hao N., Wei J., Wang K. (2022). Enhanced Photoelectrochemical Aptasensing for Sensitive Detection of Diazinon Pesticide Used N-Hydroxyphthalimide as an Effective Hole Mediator. Sens. Actuators B Chem..

[124] Zhao W.-W., Yu P.-P., Xu J.-J., Chen H.-Y. (2011). Ultrasensitive Photoelectrochemical Biosensing Based on Biocatalytic Deposition. Electrochem. Commun..

[125] Zhang L., Shi X.-M., Xu Y.-T., Fan G.-C., Yu X.-D., Liang Y.-Y., Zhao W.-W. (2019). Binding-Induced Formation of DNAzyme on an Au@Ag Nanoparticles/TiO_2_ Nanorods Electrode: Stimulating Biocatalytic Precipitation Amplification for Plasmonic Photoelectrochemical Bioanalysis. Biosens. Bioelectron..

[126] Zhu X., Gao L., Tang L., Peng B., Huang H., Wang J., Yu J., Ouyang X., Tan J. (2019). Ultrathin PtNi Nanozyme Based Self-Powered Photoelectrochemical Aptasensor for Ultrasensitive Chloramphenicol Detection. Biosens. Bioelectron..

[127] Zheng H., Zhang S., Yuan J., Qin T., Li T., Sun Y., Liu X., Wong D.K.Y. (2022). Amplified Detection Signal at a Photoelectrochemical Aptasensor with a Poly(Diphenylbutadiene)-BiOBr Heterojunction and Au-Modified CeO_2_ Octahedrons. Biosens. Bioelectron..

[128] Tan J., Peng B., Tang L., Feng C., Wang J., Yu J., Ouyang X., Zhu X. (2019). Enhanced Photoelectric Conversion Efficiency: A Novel h-BN Based Self-Powered Photoelectrochemical Aptasensor for Ultrasensitive Detection of Diazinon. Biosens. Bioelectron..

[129] Li H., Qiao Y., Li J., Fang H., Fan D., Wang W. (2016). A Sensitive and Label-Free Photoelectrochemical Aptasensor Using Co-Doped ZnO Diluted Magnetic Semiconductor Nanoparticles. Biosens. Bioelectron..

[130] Jiang D., Du X., Zhou L., Li H., Wang K. (2017). New Insights toward Efficient Charge-Separation Mechanism for High-Performance Photoelectrochemical Aptasensing: Enhanced Charge-Carrier Lifetime via Coupling Ultrathin MoS_2_ Nanoplates with Nitrogen-Doped Graphene Quantum Dots. Anal. Chem..

[131] Xiao W., Wang L., Wei X., Li J. (2022). Chitosan-Based Molecularly Imprinted Photoelectric Sensor with ZnO/Bi_2_O_3_/Bi_2_S_3_ Sensing Layer for Thiamethoxam Determination. Microchim. Acta.

[132] Shi X., Li X., Wei X., Li J. (2020). Molecularly Imprinted Photoelectrochemical Sensor Based on AgBiS_2_/Bi_2_S_3_ for Determination of Propoxur. Chin. J. Anal. Chem..

[133] Sun X., Gao C., Zhang L., Yan M., Yu J., Ge S. (2017). Photoelectrochemical Sensor Based on Molecularly Imprinted Film Modified Hierarchical Branched Titanium Dioxide Nanorods for Chlorpyrifos Detection. Sens. Actuators B Chem..

[134] Wang J., Xu Q., Xia W.W., Shu Y., Jin D., Zang Y., Hu X. (2018). High Sensitive Visible Light Photoelectrochemical Sensor Based on In-Situ Prepared Flexible Sn_3_O_4_ Nanosheets and Molecularly Imprinted Polymers. Sens. Actuators B Chem..

[135] Cao Y., Wang L., Wang C., Hu X., Liu Y., Wang G. (2019). Sensitive Detection of Glyphosate Based on a Cu-BTC MOF/g-C_3_N_4_ Nanosheet Photoelectrochemical Sensor. Electrochim. Acta.

[136] Luo Y., Mi Y., Tan X., Chen Q., Feng D., Ai C. (2019). Ultrathin BiOCl Nanosheet Modified TiO_2_ for the Photoelectrochemical Sensing of Chlorpyrifos. Anal. Methods.

[137] Liu Q., Yin Y., Hao N., Qian J., Li L., You T., Mao H., Wang K. (2018). Nitrogen Functionlized Graphene Quantum Dots/3D Bismuth Oxyiodine Hybrid Hollow Microspheres as Remarkable Photoelectrode for Photoelectrochemical Sensing of Chlopyrifos. Sens. Actuators B Chem..

[138] Wang H., Liang D., Xu Y., Liang X., Qiu X., Lin Z. (2021). A Highly Efficient Photoelectrochemical Sensor for Detection of Chlorpyrifos Based on 2D/2D β-Bi_2_O_3_/g-C_3_N_4_ Heterojunctions. Environ. Sci. Nano.

[139] Wang H., Zhang B., Zhao F., Zeng B. (2018). One-Pot Synthesis of N-Graphene Quantum Dot-Functionalized I-BiOCl Z-Scheme Cathodic Materials for “Signal-Off” Photoelectrochemical Sensing of Chlorpyrifos. ACS Appl. Mater. Interfaces.

[140] Zhang L., Feng L., Jiang J., Li P., Chen X., Zhang S., Gao Y., Hong R., Chen G., Mao G. (2021). A Highly Sensitive and Visible-Light-Driven Photoelectrochemical Sensor for Chlorpyrifos Detection Using Hollow Co9S8@CdS Heterostructures. Sens. Actuators B Chem..

[141] Chen S.-H., Xiao X.-Y., Li P.-H., Li Y.-X., Yang M., Guo Z., Huang X.-J. (2020). A Direct Z-Scheme ZnS/Co_9_S_8_ Heterojunction-Based Photoelectrochemical Sensor for the Highly Sensitive and Selective Detection of Chlorpyrifos. Environ. Sci. Nano.

[142] Zhao J., Cheng J., Sun Y., Liu J., Chen W., Xu Y., Yang J., Li Y. (2021). A Photoelectrochemical Sensor Based on Z-Scheme TiO_2_@Au@CdS and Molecularly Imprinted Polymer for Uric Acid Detection. Microchim. Acta.

[143] Limthin D., Leepheng P., Tunhoo B., Onlaor K., Klamchuen A., Phromyothin D., Thiwawong T. (2023). Preparation of Surface-Modified Electrode of Copper(II) Oxide Mixed with the Molecularly Imprinted Polymer for Enhancement of Melamine Detection with Photoelectrochemical Technique. RSC Adv..

[144] Li H., Li J., Xu Q., Yang Z., Hu X. (2013). A Derivative Photoelectrochemical Sensing Platform for 4-Nitrophenolate Contained Organophosphates Pesticide Based on Carboxylated Perylene Sensitized Nano-TiO_2_. Anal. Chim. Acta.

[145] Shi J.-J., Wang Y., Meng L.-R., Zhu J.-C., Shu R.-W., He J. (2018). Synthesis of rGO/TiO_2_/CdS Nanocomposites and Its Enhanced Photoelectrochemical Performance in Determination of Parathion-Methyl. Nano.

[146] Yan Y., Li Q., Wang Q., Mao H. (2021). A One-Step Hydrothermal Route to Fabricate a ZnO Nanorod/3D Graphene Aerogel-Sensitized Structure with Enhanced Photoelectrochemistry Performance and Self-Powered Photoelectrochemical Biosensing of Parathion-Methyl. RSC Adv..

[147] Cheng W., Zheng Z., Yang J., Chen M., Yao Q., Chen Y., Gao W. (2019). The Visible Light-Driven and Self-Powered Photoelectrochemical Biosensor for Organophosphate Pesticides Detection Based on Nitrogen Doped Carbon Quantum Dots for the Signal Amplification. Electrochim. Acta.

[148] Du D., Ding J., Tao Y., Chen X. (2008). Application of Chemisorption/Desorption Process of Thiocholine for Pesticide Detection Based on Acetylcholinesterase Biosensor. Sens. Actuators B Chem..

[149] Wang M., Hou L., Chen X., Lin T. (2022). Homogeneous Photoelectrochemical Biosensor for Sensitive Detection of Omethoate via ALP-Mediated Pesticide Assay and Bi_2_S_3_@Bi_2_Sn_2_O_7_ Heterojunction as Photoactive Material. Anal. Bioanal. Chem..

[150] Jin D., Gong A., Zhou H. (2017). Visible-Light-Activated Photoelectrochemical Biosensor for the Detection of the Pesticide Acetochlor in Vegetables and Fruit Based on Its Inhibition of Glucose Oxidase. RSC Adv..

[151] Zeng Z., Tang J., Zhang M., Pu S., Tang D. (2021). Ultrasensitive Zero-Background Photoelectrochemical Biosensor for Analysis of Organophosphorus Pesticide Based on in Situ Formation of DNA-Templated Ag_2_S Photoactive Materials. Anal. Bioanal. Chem..

[152] Li J., Xiong P., Tang J., Liu L., Gao S., Zeng Z., Xie H., Tang D., Zhuang J. (2021). Biocatalysis-Induced Formation of BiOBr/Bi_2_S_3_ Semiconductor Heterostructures: A Highly Efficient Strategy for Establishing Sensitive Photoelectrochemical Sensing System for Organophosphorus Pesticide Detection. Sens. Actuators B Chem..

[153] Tian J., Li Y., Dong J., Huang M., Lu J. (2018). Photoelectrochemical TiO_2_ Nanotube Arrays Biosensor for Asulam Determination Based on In-Situ Generation of Quantum Dots. Biosens. Bioelectron..

[154] Qin Y., Wu Y., Chen G., Jiao L., Hu L., Gu W., Zhu C. (2020). Dissociable Photoelectrode Materials Boost Ultrasensitive Photoelectrochemical Detection of Organophosphorus Pesticides. Anal. Chim. Acta.

[155] Tang J., Li J., Xiong P., Sun Y., Zeng Z., Tian X., Tang D. (2020). Rolling Circle Amplification Promoted Magneto-Controlled Photoelectrochemical Biosensor for Organophosphorus Pesticides Based on Dissolution of Core-Shell MnO_2_ nanoflower@CdS Mediated by Butyrylcholinesterase. Microchim. Acta.

[156] Liu Q., Huan J., Dong X., Qian J., Hao N., You T., Mao H., Wang K. (2016). Resonance Energy Transfer from CdTe Quantum Dots to Gold Nanorods Using MWCNTs/rGO Nanoribbons as Efficient Signal Amplifier for Fabricating Visible-Light-Driven “on-off-on” Photoelectrochemical Acetamiprid Aptasensor. Sens. Actuators B Chem..

[157] Xu B.-F., Li Q., Qu P., Xin X.-R., Wang A.-J., Mei L.-P., Song P., Feng J.-J. (2023). Magnetic-Assisted Exciton-Plasmon Interactions Modulated Bi_2_S_3_ nanorods@MoS_2_ Nanosheets Heterojunction: Towards a Split-Type Photoelectrochemical Sensing of Profenofos. Microchim. Acta.

[158] Wang G., Li L., Zheng H., Li Q., Huang J., Zhang L., Yang H., Cui K., Yu J. (2023). Bifunctional Strategy toward Constructing Perovskite/Upconversion Lab-on-Paper Photoelectrochemical Device for Sensitive Detection of Malathion. ACS Nano.

